# Effect of a one-time financial incentive on linkage to chronic hypertension care in Kenya and Uganda: A randomized controlled trial

**DOI:** 10.1371/journal.pone.0277312

**Published:** 2022-11-07

**Authors:** Matthew D. Hickey, Asiphas Owaraganise, Norton Sang, Fredrick J. Opel, Erick Wafula Mugoma, James Ayieko, Jane Kabami, Gabriel Chamie, Elijah Kakande, Maya L. Petersen, Laura B. Balzer, Moses R. Kamya, Diane V. Havlir

**Affiliations:** 1 Division of HIV, Infectious Disease, & Global Medicine, University of California, San Francisco, CA, United States of America; 2 Infectious Diseases Research Collaboration, Kampala, Uganda; 3 Kenya Medical Research Institute, Nairobi, Kenya; 4 School of Public Health, University of California, Berkeley, CA, United States of America; 5 School of Medicine, Makerere University, Kampala, Uganda; KEMRI Wellcome Trust Research Programme, KENYA

## Abstract

**Background:**

Fewer than 10% of people with hypertension in sub-Saharan Africa are diagnosed, linked to care, and achieve hypertension control. We hypothesized that a one-time financial incentive and phone call reminder for missed appointments would increase linkage to hypertension care following community-based screening in rural Uganda and Kenya.

**Methods:**

In a randomized controlled trial, we conducted community-based hypertension screening and enrolled adults ≥25 years with blood pressure ≥140/90 mmHg on three measures; we excluded participants with known hypertension or hypertensive emergency. The intervention was transportation reimbursement upon linkage (~$5 USD) and up to three reminder phone calls for those not linking within seven days. Control participants received a clinic referral only. Outcomes were linkage to hypertension care within 30 days (primary) and hypertension control <140/90 mmHg measured in all participants at 90 days (secondary). We used targeted minimum loss-based estimation to compute adjusted risk ratios (aRR).

**Results:**

We screened 1,998 participants, identifying 370 (18.5%) with uncontrolled hypertension and enrolling 199 (100 control, 99 intervention). Reasons for non-enrollment included prior hypertension diagnosis (n = 108) and hypertensive emergency (n = 32). Participants were 60% female, median age 56 (range 27–99); 10% were HIV-positive and 42% had baseline blood pressure ≥160/100 mmHg. Linkage to care within 30 days was 96% in intervention and 66% in control (aRR 1.45, 95%CI 1.25–1.68). Hypertension control at 90 days was 51% intervention and 41% control (aRR 1.22, 95%CI 0.92–1.66).

**Conclusion:**

A one-time financial incentive and reminder call for missed visits resulted in a 30% absolute increase in linkage to hypertension care following community-based screening. Financial incentives can improve the critical step of linkage to care for people newly diagnosed with hypertension in the community.

## Introduction

Hypertension is the largest driver of morbidity and mortality globally and can be treated with low-cost, readily available medications; however, less than 10% of people with hypertension in sub-Saharan Africa are diagnosed, linked to care, and have controlled hypertension [1–4]. Community-based diagnosis and linkage to care are critical steps to improving hypertension control in sub-Saharan Africa, however, linkage to care following diagnosis has not surpassed 50% in several studies [5–7].

High transportation costs, long distances from clinic, opportunity costs associated with missed work, and medication costs may all contribute to low rates of linkage to care [8–10]. Lack of awareness of the consequences of hypertension or competing priorities may also reduce motivation to seek hypertension care [11]. Incentives may address both financial barriers and provide extrinsic motivation to overcome initial barriers to engaging with hypertension care. Promising findings from a prior single-arm pilot study showed very high levels of linkage to care with a small conditional financial incentive at a single site in Uganda [12].

Though literature on incentives for linkage to hypertension care in sub-Saharan Africa is limited, there is evidence from the HIV literature that financial incentives are effective for improving HIV testing and to a lesser extent linkage to HIV care [13]. McNairy et al. showed that a combination intervention including a nonfinancial incentive (mobile airtime) increased joint linkage and retention in care after 12 months following a new HIV diagnosis [14]. We have also shown high levels of linkage to HIV care following community-based screening with a combination intervention including introduction to a clinic staff member, provision of a “hot line” to answer questions about linkage, a small transportation reimbursement conditional upon linkage, and reminder calls for missed linkage appointments [15].

Our study was motivated by the very low levels of linkage to hypertension care seen in other studies of hypertension linkage interventions in sub-Saharan Africa to date and the promising data from the HIV literature on effectiveness of one-time incentives for improving linkage to care. We conducted a randomized controlled trial to test the hypothesis that a one-time financial incentive and phone call reminder for missed appointments would increase linkage to hypertension care following community-based screening in rural Uganda and Kenya.

## Methods

### Setting and participants

Between April and June 2021, we conducted community-based hypertension screening in two rural communities in western Kenya and one rural community in southwestern Uganda. A study nurse and local lay health workers conducted the screening at central locations in the community (e.g. markets, places of worship, schools) and through household screening when COVID-19 measures precluded screening at community gathering points.

Participants were eligible if they were ≥25 years of age with elevated blood pressure (BP) on community-based screening (systolic blood pressure [SBP] ≥140 mmHg or diastolic blood pressure [DBP] ≥90 mmHg on each of three measurements performed by a trained study nurse using standardized procedures). BP was measured once using an automated BP machine after the participant had been seated for five minutes; those with SBP ≥140 or DBP ≥90 mmHg had two additional measurements taken at 1–2 minute intervals. Participants were excluded if they had a prior known diagnosis of hypertension, current pregnancy, or hypertensive emergency (BP ≥180/110 mmHg and signs/symptoms of target organ damage). Persons with a known diagnosis of hypertension were excluded because the intervention was designed to evaluate first-time linkage to hypertension care, rather than re-engagement. Pregnant women with hypertension or those with hypertensive emergency were immediately transported to a health facility for further management. Those with known hypertension were counseled on risks of untreated hypertension and referred to re-engage in hypertension care if needed. The study received Institutional Review Board approval from Makerere University, the Kenya Medical Research Institute, and the University of California San Francisco. Participants provided written informed consent prior to study participation. The study is registered at ClincialTrials.gov (NCT04810650).

### Study design and procedures

We conducted an individually randomized controlled trial of a one-time financial incentive and phone reminder calls to facilitate linkage to hypertension care among eligible participants with uncontrolled hypertension. Eligible participants were randomized to the intervention or control by selecting a sequentially numbered scratch card, revealing the arm only when scratched by the participant. The computer-generated randomization sequence was stratified on site and sex and implemented with a stratified block design with random block sizes of 2 and 4. Participants were not blinded to the randomization arm, but the study statistician (LBB) was blinded until trial completion and analysis. Based on a two-sample test for proportions, we estimated 100 participants/arm would provide 80% power to detect a 20% absolute increase in the primary outcome of linkage within 30 days from 50% in the control.

All participants received post-test counseling and were scheduled for an initial hypertension care visit at a nearby government health facility within three days if BP ≥160/100mmHg and within seven days if BP was 140-159/90-99. Intervention participants received a transportation voucher and were informed that the voucher was redeemable for ~$5 USD upon linkage to hypertension care. If intervention participants did not link to care by their scheduled appointment date, they received up to three phone call attempts to encourage linkage. Control participants did not receive a linkage incentive or follow-up phone calls.

Hypertension care was delivered at Ministry of Health clinics within each community using country standard hypertension guidelines [16, 17] and a patient-centered HIV care model with multi-disease integration (chronic HIV, hypertension, and/or diabetes care) for both intervention and control participants [5]. Care was provided in collaboration between study clinicians and Ministry of Health clinicians at each government clinic. Medications were procured by the clinics and were supplemented by the study when clinic-procured medications were not available. All medications were provided to participants free of charge.

### Measurements

We conducted baseline surveys (i.e., at time of community-based hypertension screening) to record participant demographics, medical co-morbidities, and perceived barriers and facilitators of hypertension care engagement. Barriers and facilitators were assessed with an open-ended question about factors that would make it easier/more difficult to go to the clinic for hypertension care. Responses were coded with a structured set of answer choices that could be used as prompts when participants were unsure. We recorded clinical data from all hypertension care visits, including date of visit, blood pressure measured, and medications prescribed.

At three months post-enrollment, we conducted follow-up study visits for all participants, regardless of linkage to hypertension care. We collected data on self-reported linkage to any alternative clinical sites for hypertension care and any barriers/facilitators that participants experienced to accessing hypertension care using the same method described above. We measured blood pressure, conducting three measures in all participants using an automated BP machine and correctly sized cuff after the participant had been seated quietly with back and arm supported for five minutes, with repeat measurements made after 1–2 minute intervals. Hypertension was considered controlled if all three measures were <140 mmHg systolic and <90 mmHg diastolic.

### Outcomes and analysis

Our primary outcome was linkage to hypertension care at the local Ministry of Health clinic within 30 days of referral. In primary analyses, participants without a clinical record indicating linkage were assumed never to link, while sensitivity analyses incorporated self-report. Secondary outcomes included hypertension control <140/90 mmHg measured regardless of linkage at 90 days, linkage to any hypertension care by 90 days (linkage to Ministry of Health clinic or self-report of linkage to alternative site), retention in hypertension care at 90 days (linked to hypertension care and not late for most recent appointment by ≥30 days). We additionally describe medication initiation by trial arm and fidelity to intervention components. For primary and secondary outcomes, we used targeted minimum loss-based estimation (TMLE) to compute adjusted risk ratios (aRR) and 95% confidence intervals (95% CI) (S1 File) [18, 19]. In brief, TMLE combines estimates of outcome risk with estimates of propensity score and incorporates adjustment for baseline covariates to improve precision [20–22]. We selected adjustment variables (candidates were age, sex, hypertension severity, and site) with cross-validation. We also compared by arm the average of the second and third blood pressure measurements. To explore heterogeneity of intervention effects, we also repeated analyses within subgroups defined by sex, age group, country, baseline hypertension severity, and HIV status.

## Results

**Fig 1:**
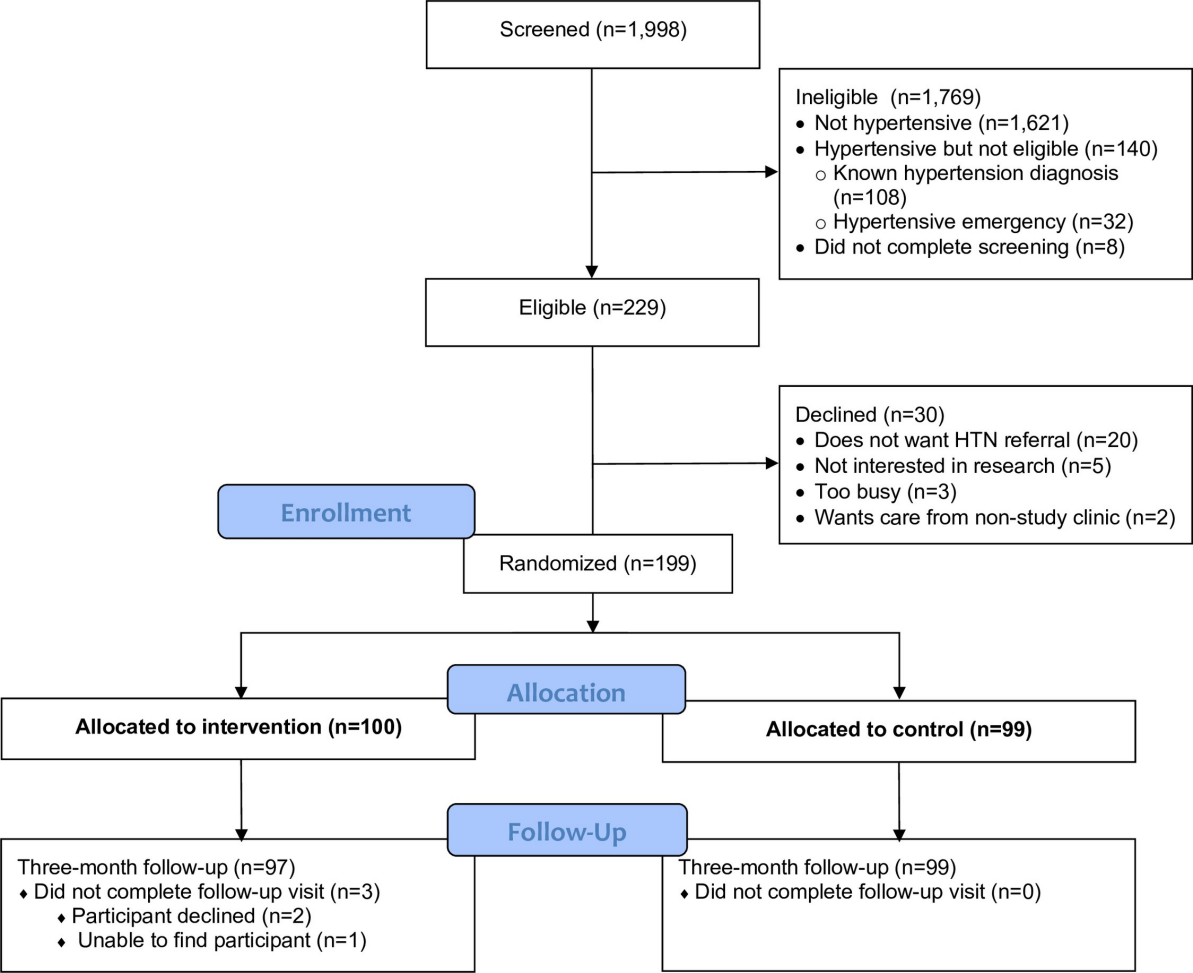
Consort diagram.

We screened 1,998 participants, identifying 369 (18.5%) with uncontrolled hypertension. Among 229 eligible persons, we enrolled 199 (100 control, 99 intervention; Fig 1). Reasons for ineligibility included prior hypertension diagnosis (n = 108) and hypertensive emergency (n = 32). People with hypertensive emergency at screening were immediately transported to the clinic for treatment. Among those with hypertensive emergency, mean SBP was 198 mmHg (standard deviation [SD] 15) and mean DBP was 104 mmHg (SD 12); symptoms included visual disturbances (n = 18, 56%), headache (n = 16, 50%), chest pain (n = 11, 34%), shortness of breath (n = 9, 28%) and recent lapse in consciousness (n = 6, 19%).

Of the enrolled participants 60% were female, median age was 56 (range 27–99) years, and 87% had completed primary education or less (Table 1). Mean baseline SBP was 161 mmHg (SD 14) and 42% had baseline blood pressure ≥160/100 mmHg. Median self-reported direct cost of transport to the clinic was $0.93 USD (range $0.19 to $4.67) and median transport time was 45 minutes (range 2 to 300 minutes). Self-reported co-morbid conditions included HIV (n = 19, 10%) and diabetes (n = 3, 2%). HIV screening was offered to all participants at baseline; 100 (50%) were tested, among whom one participant was newly diagnosed with HIV. Reasons for declining HIV testing included known HIV-positive status (n = 19), having a negative test in the past 6 months and thus not meeting country guidelines for retesting (n = 41), declining testing (n = 14), and never linking to the clinic to offer HIV testing (n = 26).

**Table 1:** Baseline characteristics.

	Intervention (N = 100) n (%)	Control (N = 99) n (%)	Overall (N = 199) n (%)	p-value
**Country**				1.00
Kenya	50 (50.0)	49 (49.5)	99 (49.7)	
Uganda	50 (50.0)	50 (50.5)	100 (50.3)	
**Age (years)**				0.63
25–39	15 (15.0)	16 (16.2)	31 (15.6)	
40–54	28 (28.0)	31 (31.3)	59 (29.6)	
55–69	36 (36.0)	39 (39.4)	75 (37.7)	
70–84	18 (18.0)	12 (12.1)	30 (15.1)	
>84	3 (3.0)	1 (1.0)	4 (2.0)	
**Sex**				1.00
Female	60 (60.0)	59 (59.6)	119 (59.8)	
Male	40 (40.0)	40 (40.4)	80 (40.2)	
**Marital status**				0.67
Monogamous marriage	58 (58.0)	56 (56.6)	114 (57.3)	
Polygamous marriage	17 (17.0)	21 (21.2)	39 (19.6)	
Widowed, divorced, or separated	22 (22.0)	17 (17.0)	38 (19.1)	
Unmarried or cohabitating	3 (3.0)	6 (6.0)	8 (4.0)	
**Occupation**				0.62
Farmer	71 (71.0)	64 (64.6)	135 (67.8)	
No job	8 (8.0)	7 (7.1)	15 (7.5)	
Teacher	3 (3.0)	5 (5.1)	8 (4.0)	
Other	18 (18.0)	23 (23.2)	32 (16.1)	
**Monthly Income (USD)**				
Mean (SD)	21 (34)	27 (29)	24 (32)	0.27
Median (Q1-Q3)	11 (5.7–23)	17 (6.6–37)	11 (5.7–34)	
Missing	16 (16.0)	16 (16.2)	32 (16.1)	
**Transport cost to clinic (USD)**				
Mean (SD)	1.27 (0.70)	1.12 (0.54)	1.19 (0.63)	0.10
Median (Q1-Q3)	1.1 (0.86–1.4)	0.93 (0.86–1.4)	0.93 (0.86–1.4)	
**Mobile phone access**				0.69
No	14 (14.0)	16 (16.2)	30 (15.1)	
Yes	86 (86.0)	83 (83.8)	169 (84.9)	
**Education**				0.78
Less than primary	55 (55.0)	62 (62.6)	117 (58.8)	
Completed primary	32 (32.0)	25 (25.3)	57 (28.6)	
Completed secondary	3 (3.0)	3 (3.0)	6 (3.0)	
Completed post-secondary	10 (10.0)	9 (9.1)	19 (9.5)	
**Baseline diabetes**	2 (2.0)	1 (1.0)	3 (1.5)	0.56
Baseline HIV*	6 (6.0)	14 (14.1)	20 (10.0)	0.06
**Baseline Hypertension Stage (mmHg)**				0.93
140-159/90-99	58 (58.0)	58 (58.6)	116 (58.3)	
160-179/100-109	38 (38.0)	38 (38.4)	76 (38.2)	
≥180/110	4 (4.0)	3 (3.0)	7 (3.5)	
**SBP at baseline (mmHg)**				
Mean (SD)	160 (13.1)	161 (14.8)	161 (13.9)	0.77
Median (Q1-Q3)	157 (149–169)	158 (147–171)	158 (149–170)	

* Including self-report and one new diagnosis on baseline screening.

### Primary and secondary outcomes

Linkage to care within 30 days was 96/100 (96%) in intervention and 65/99 (66%) in control, a relative improvement of 45% (aRR 1.45, 95% CI 1.25–1.68; Table 2) and an absolute improvement of 30% (risk difference 0.30, 95% CI 0.20–0.40). The intervention improved linkage to care within 30 days across strata of age, gender, country, baseline hypertension severity, and HIV status (Table 3). The largest effect was seen among those with baseline blood pressure ≥160/100 mmHg, among whom the intervention increased linkage by 69% (aRR 1.69, 95% CI 1.31–2.18). After expanding the period to 90 days and incorporating self-reported any health facility, linkage remained at 96% in the intervention and increased to 77% in the control group (n = 74 at local Ministry of Health clinic and n = 2 to other self-reported clinic) (aRR 1.24, 95% CI 1.11–1.40).

**Table 2:** Main study outcomes.

	Intervention (N = 100) n (%)	Control (N = 99) n (%)	aRR (95% CI)
**Linked to care by 30 days**	96 (96)	65 (66)	1.45 (1.25, 1.68)
Linked to care by 3 months *	96 (96)	77 (78)	1.24 (1.11, 1.39)
**Retained in care at 3 months †**	78 (78)	67 (68)	1.15 (0.97, 1.37)
**Started on ≥1 hypertension medication ‡**			
Any	48 (48)	40 (40)	
1	20 (20)	18 (18)	
2	26 (26)	21 (21)	
3	2 (2)	1 (1)	
**Hypertension control at 3 months**	51 (51)	41 (41)	1.23 (0.92, 1.66)
**Mean SBP at 3 months**	140	141	0.99 (0.96, 1.02)

* including self-reported linkage to alternative clinic.

† not late for most recent hypertension clinic visit by ≥30 days (assessed at 3 months post-enrollment).

‡ Outcome not prespecified and no between arm comparison undertaken.

**Table 3:** Linkage to care within 30 days, stratified by baseline participant characteristics.

	Intervention (N = 100)	Control (N = 99)	aRR (95% CI)
**Gender**			
Men	0.95	0.67	1.41 (1.12, 1.77)
Women	0.97	0.65	1.49 (1.24, 1.81)
**Age**			
< 60 years	0.97	0.62	1.56 (1.27, 1.92)
≥60 years	0.95	0.72	1.32 (1.08, 1.60)
**Country**			
Uganda	0.94	0.59	1.58 (1.25, 2.00)
Kenya	0.98	0.72	1.36 (1.14, 1.63)
**Baseline Hypertension Stage**			
140-159/90-99 mmHg	0.93	0.71	1.32 (1.1, 1.58)
≥160/100 mmHg	1	0.59	1.69 (1.31, 2.18)
**Baseline HIV status**			
HIV-positive	1	0.8	1.26 (0.96, 1.65)
HIV-negative	0.96	0.64	1.49 (1.27, 1.76)

Hypertension control <140/90 mmHg at three months was measured independently from clinical care in 96/100 (96%) of intervention participants and 99/99 (100%) in control); participants with missing measures were assumed uncontrolled. Hypertension control was 51/100 (51%) in intervention and 41/99 (41%) in control (aRR 1.23, 95% CI 0.92–1.66). Among persons with baseline blood pressure ≥160/100 mmHg, hypertension control was 46% in intervention and 26% in control (aRR 1.78, 95% CI 0.98–3.22; Table 4). Mean SBP at three months was 140 mmHg in intervention and 141 mmHg in control.

**Table 4:** Hypertension control at 3 months, stratified by baseline participant characteristics.

	Intervention (N = 100)	Control (N = 99)	aRR (95% CI)
**Gender**			
Men	0.46	0.41	1.13 (0.69, 1.84)
Women	0.55	0.42	1.32 (0.91, 1.93)
**Age**			
< 60 years	0.51	0.41	1.23 (0.84, 1.82)
≥60 years	0.49	0.44	1.09 (0.71, 1.69)
**Country**			
Uganda	0.42	0.3	1.4 (0.82, 2.38)
Kenya	0.6	0.54	1.12 (0.80, 1.58)
**Baseline Hypertension Stage**			
140-159/90-99 mmHg	0.52	0.55	0.95 (0.69, 1.30)
≥160/100 mmHg	0.46	0.26	1.78 (0.98, 3.22)
**Baseline HIV status**			
HIV-positive	0.68	0.49	1.39 (0.64, 3.03)
HIV-negative	0.49	0.42	1.14 (0.85, 1.53)

### Intervention fidelity and delivery of clinical hypertension care

All intervention participants received transport reimbursement upon linkage. Of intervention participants who did not link to care by seven days and were eligible for a reminder call (n = 23), 13 (56%) were successfully reached by phone, one (4%) was reached via an informant, six (26%) linked to care before a phone call could be initiated, and three (13%) could not be reached by phone. Among those who did not link to care by their initial clinic appointment date (scheduled within seven days of enrollment), 83% (n = 19/23) of intervention participants and 26% (n = 12/46) of control participants ultimately linked to hypertension care by 30 days post-enrollment.

Among those who linked to care (n = 175), 50 (29%) had normal blood pressure (<140/90 mmHg) on their initial clinic visit, 88 (50%) had stage 1 hypertension (BP 140-159/90-99 mmHg), and 37 (21%) had stage 2 or greater hypertension (BP ≥160/100 mmHg). Among those with stage 1 hypertension, 58% received lifestyle counseling and 42% were immediately initiated on antihypertensive medications. Among those with stage 2 or greater hypertension, 84% were initiated on antihypertensive medications at the first clinic visit. After three months, 48% (n = 48) of intervention participants and 40% (n = 40) of control participants had started at least one antihypertensive medication. Seventy-eight (78%) intervention participants and 67 (68%) control participants were retained in care by three months (aRR 1.15, 95% CI 0.97–1.37). In sub-group analysis, the intervention increased retention in care in women (aRR 1.34, 95% CI 1.06–1.70), Uganda (aRR 1.55, 95% CI 1.19–2.0), among those with grade 2 or greater baseline hypertension stage (aRR 1.32, 95% CI 1.02–1.70), and those who were HIV negative at baseline (aRR 1.17, 95% CI 0.98–1.4) (Table 5).

**Table 5:** Retention in care at 3 months, stratified by baseline participant characteristics.

	Intervention (N = 100)	Control (N = 99)	aRR (95% CI)
**Gender**			
Men	0.73	0.77	0.96 (0.75, 1.22)
Women	0.82	0.61	1.34 (1.06, 1.70)
**Age**			
< 60 years	0.78	0.7	1.11 (0.89, 1.38)
≥60 years	0.79	0.64	1.22 (0.93, 1.61)
**Country**			
Uganda	0.86	0.56	1.55 (1.19, 2.03)
Kenya	0.7	0.8	0.88 (0.7, 1.11)
**Baseline Hypertension Stage**			
140-159/90-99 mmHg	0.74	0.72	1.02 (0.82, 1.28)
≥160/100 mmHg	0.84	0.64	1.32 (1.02, 1.70)
**Baseline HIV status**			
HIV-positive	0.81	0.8	1.02 (0.67, 1.57)
HIV-negative	0.78	0.67	1.17 (0.98, 1.40)

### Self-reported barriers and facilitators of hypertension care engagement

**Fig 2:**
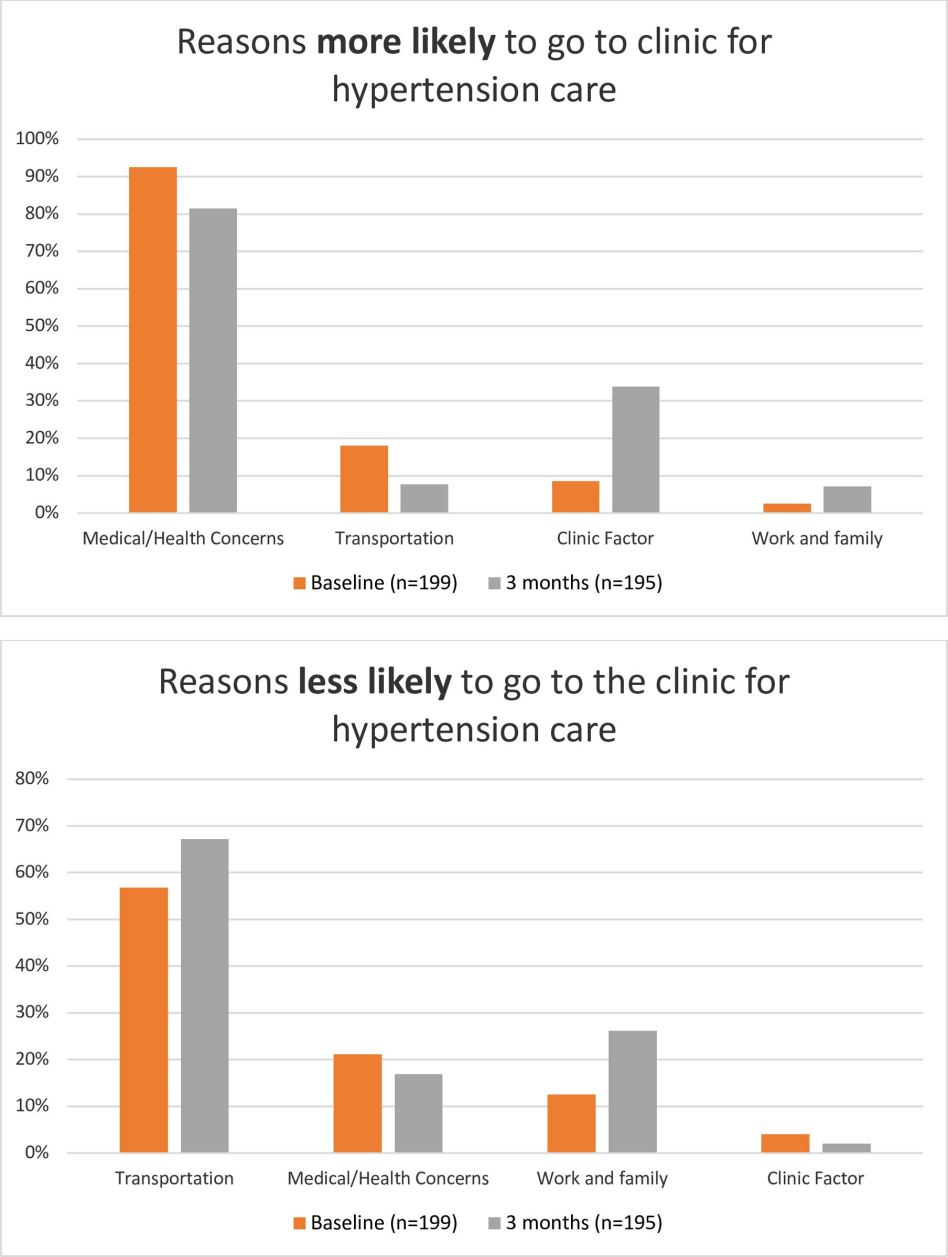
Self-reported barriers and facilitators of engagement in hypertension care.

At baseline, 92% of participants cited health concerns as reasons why they were likely to seek hypertension care (61% cited worry about their hypertension diagnosis and 43% cited feeling sick and wanting to seek care). Over half (57%) cited transportation difficulties as reasons why they were less likely to seek hypertension care (50% cited transportation expense and 11% cited long distance or difficulties with physical mobility) (Fig 2).

## Discussion

Following community-based hypertension screening, a one-time financial incentive and reminder call for missed visits resulted in a 30% absolute increase in linkage to hypertension care within 30 days, compared to standard referrals. Increased linkage to care translated to a greater proportion of participants achieving hypertension control <140/90 mmHg in the intervention arm, though overall hypertension control remained sub-optimal.

Across sub-Saharan Africa, an estimated 27% of adults with hypertension are aware of their diagnosis, with only 18% currently on treatment, and 7% with controlled hypertension [2]. Limitations in diagnosis and treatment are similar in East Africa [1, 3, 23], even among those at greatest CVD risk [24]. We previously showed that patient-centered hypertension care can improve hypertension control and reduce all-cause mortality among adults with uncontrolled hypertension identified through population-level screening; however, fewer than half of patients diagnosed with hypertension linked to care within one year, limiting the full benefits of treatment [5, 25]. Other studies have identified similar challenges linking patients to hypertension care following community-based screening. In the LARK cluster randomized controlled trial in Kenya, linkage to care within a year was 49% following community-based screening and referral to hypertension by community health workers (CHWs), with no added improvement in linkage achieved by training CHWs in behavioral communication and providing smartphones to facilitate health communication [6]. A mobile multi-disease screening intervention in South Africa also showed similar rates of linkage to hypertension care of approximately 50% [7].

Incentives may improve linkage to care by reducing economic barriers, addressing present bias, and increasing extrinsic motivation [26]. In our study, self-reported round trip transportation costs for a single clinic visit were approximately 8% of median monthly wages, and transportation time/cost was the most commonly reported reason for not seeking hypertension care. The size of our one-time incentive was approximately 5-fold greater than median transportation costs with the goal of directly reimbursing transportation costs, addressing opportunity costs associated with clinic attendance, and providing extrinsic motivation. A recent RCT in Kenya showed small, non-significant reductions in systolic blood pressure when coupling hypertension care with a microfinance intervention designed to improve financial stability, suggesting that interventions to address poverty may address some of the financial barriers to care engagement [27]. However, adding a conditional incentive to link to care may increase linkage more than a similarly valued non-conditional incentive, highlighting the important role of extrinsic motivation in linkage interventions, particularly for medical conditions, such as hypertension, where early-stage disease may be asymptomatic [28].

Our incentives were not targeted towards any particular group, though evidence from the HIV literature also demonstrates that incentives may be particularly useful for promoting uptake of health services among groups who are less likely to seek healthcare services, particularly men [29–31]. Our one-time incentive had similar effects on linkage among men and women. However, once the incentive was no longer available (i.e. for follow-up visits), there was no sustained intervention effect on blood pressure control or retention in care among men. Future iterations of our intervention may consider a more targeted approach towards individuals who are less likely to engage with hypertension care on their own or who may be at higher risk of adverse outcomes if they do not link promptly.

Our intervention was designed to overcome initial barriers to linkage to care but did not address ongoing transportation challenges that patients may face. Nonetheless, higher linkage to care in the intervention arm translated to higher short-term retention in care, suggesting that “getting patients in the door” may overcome the initial activation energy required to ensure consistent engagement in care, at least in the short term. Upon linkage, patients in both treatment arms received integrated, patient-centered hypertension care [5] and free antihypertensive medications, which likely attenuated differences in retention and hypertension control between arms. After three months, one-third of participants cited clinic factors as reasons for remaining in hypertension care (i.e. high quality of care, short waiting time, kind staff, and availability of medications), highlighting the value of high-quality care in encouraging retention.

Our pilot study had several important limitations. First, follow-up time was short and may not have been enough time to see the full effect of antihypertensive medication up-titration or to understand whether intervention effects are sustained over time. Further, 30% of participants did not have sustained elevation of blood pressure on linkage. Guidelines recommend that a diagnosis of hypertension be made after demonstrating persistent blood pressure elevation over time [16, 32, 33], therefore many of these individuals may have been overdiagnosed on initial screening. Future cost-effectiveness analysis will help quantify the resource allocation trade-offs involved in providing incentives for linkage to care for all individuals with elevated blood pressure on screening, versus limiting enhanced interventions to those with greater elevations in blood pressure. Future study evaluating smaller incentives may also be needed to address issues of scalability. As an individual-level randomized trial, there is also the possibility of contamination between trial arms if participants shared their transportation vouchers, though this would have biased study findings towards the null. Finally, our study was not designed to address linkage for individuals with hypertensive emergency. During our community-based screening, one out of every 43 individuals screened had hypertensive urgency (blood pressure ≥180/110 mmHg) or emergency (urgency + new/severe associated symptoms). Given the high morbidity and mortality of this condition in sub-Saharan Africa [34–41] new intervention approaches are needed to address the urgent and more intensive treatment needs of this population.

## Conclusions

One-time financial incentives and phone-based follow-up to ensure linkage are effective strategies for increasing linkage to hypertension care following community-based screening in rural East Africa. Diagnosis and linkage to care are critical first steps to reducing disparities in hypertension treatment outcomes in sub-Saharan Africa; health system strengthening to deliver chronic hypertension care is essential to ensuring sustained treatment benefit.

## Supporting information

S1 File Statistical analysis plan. (PDF)

S2 File Consort checklist. (DOC)

S3 File Study protocol. (DOCX)

S4 File Inclusivity in global research. (PDF)

## References

[1] Geldsetzer P, Manne-Goehler J, Marcus ME, Ebert C, Zhumadilov Z, Wesseh CS, et al. The state of hypertension care in 44 low-income and middle-income countries: a cross-sectional study of nationally representative individual-level data from 1·1 million adults. Lancet. 2019 24;394(10199):652–62. doi: 10.1016/S0140-6736(19)30955-9 31327566

[2] Ataklte F, Erqou S, Kaptoge S, Taye B, Echouffo-Tcheugui JB, Kengne AP. Burden of undiagnosed hypertension in sub-Saharan Africa: a systematic review and meta-analysis. Hypertension. 2015 Feb;65(2):291–8. doi: 10.1161/HYPERTENSIONAHA.114.04394 25385758

[3] Wamai RG, Kengne AP, Levitt N. Non-communicable diseases surveillance: overview of magnitude and determinants in Kenya from STEPwise approach survey of 2015. BMC Public Health. 2018 Nov 7;18(3):1224. doi: 10.1186/s12889-018-6051-z 30400841 PMC6218983

[4] NCD Risk Factor Collaboration (NCD-RisC). Worldwide trends in hypertension prevalence and progress in treatment and control from 1990 to 2019: a pooled analysis of 1201 population-representative studies with 104 million participants. Lancet. 2021 Sep 11;398(10304):957–80. doi: 10.1016/S0140-6736(21)01330-1 34450083 PMC8446938

[5] Hickey MD, Ayieko J, Owaraganise A, Sim N, Balzer LB, Kabami J, et al. Effect of a patient-centered hypertension delivery strategy on all-cause mortality: Secondary analysis of SEARCH, a community-randomized trial in rural Kenya and Uganda. PLoS Med. 2021 Sep 20;18(9):e1003803. doi: 10.1371/journal.pmed.1003803 34543267 PMC8489716

[6] Vedanthan R, Kamano JH, DeLong AK, Naanyu V, Binanay CA, Bloomfield GS, et al. Community Health Workers Improve Linkage to Hypertension Care in Western Kenya. J Am Coll Cardiol. 2019 Oct 15;74(15):1897–906. doi: 10.1016/j.jacc.2019.08.003 31487546 PMC6788970

[7] Govindasamy D, Kranzer K, Schaik N van, Noubary F, Wood R, Walensky RP, et al. Linkage to HIV, TB and Non-Communicable Disease Care from a Mobile Testing Unit in Cape Town, South Africa. PLOS ONE. 2013 Nov 13;8(11):e80017. doi: 10.1371/journal.pone.0080017 24236170 PMC3827432

[8] Brathwaite R, Hutchinson E, McKee M, Palafox B, Balabanova D. The Long and Winding Road: A Systematic Literature Review Conceptualising Pathways for Hypertension Care and Control in Low- and Middle-Income Countries. Int J Health Policy Manag. 2020 Jul 18. 10.34172/ijhpm.2020.105 PMC9278472 32702800

[9] Kwarisiima D, Atukunda M, Owaraganise A, Chamie G, Clark T, Kabami J, et al. Hypertension control in integrated HIV and chronic disease clinics in Uganda in the SEARCH study. BMC Public Health. 2019 May 6;19(1):511. doi: 10.1186/s12889-019-6838-6 31060545 PMC6501396

[10] Wierzejska E, Giernaś B, Lipiak A, Karasiewicz M, Cofta M, Staszewski R. A global perspective on the costs of hypertension: a systematic review. Arch Med Sci. 2020;16(5):1078–91. doi: 10.5114/aoms.2020.92689 32863997 PMC7444692

[11] Muddu M, Ssinabulya I, Kigozi SP, Ssennyonjo R, Ayebare F, Katwesigye R, et al. Hypertension care cascade at a large urban HIV clinic in Uganda: a mixed methods study using the Capability, Opportunity, Motivation for Behavior change (COM-B) model. Implement Sci Commun. 2021 Oct 20;2(1):121. doi: 10.1186/s43058-021-00223-9 34670624 PMC8690902

[12] Kotwani P, Balzer L, Kwarisiima D, Clark TD, Kabami J, Byonanebye D, et al. Evaluating linkage to care for hypertension after community-based screening in rural Uganda. Tropical medicine & international health: TM & IH. 2014 Apr;19(4):459–68. doi: 10.1111/tmi.12273 24495307 PMC4118739

[13] Koduah Owusu K, Adu-Gyamfi R, Ahmed Z. Strategies To Improve Linkage To HIV Care In Urban Areas Of Sub-Saharan Africa: A Systematic Review. HIV AIDS (Auckl). 2019;11:321–32. doi: 10.2147/HIV.S216093 31819663 PMC6898990

[14] McNairy ML, Lamb MR, Gachuhi AB, Nuwagaba-Biribonwoha H, Burke S, Mazibuko S, et al. Effectiveness of a combination strategy for linkage and retention in adult HIV care in Swaziland: The Link4Health cluster randomized trial. PLoS Med. 2017 Nov;14(11):e1002420. doi: 10.1371/journal.pmed.1002420 29112963 PMC5675376

[15] Ayieko J, Petersen ML, Charlebois ED, Brown LB, Clark TD, Kwarisiima D, et al. A Patient-Centered Multicomponent Strategy for Accelerated Linkage to Care Following Community-Wide HIV Testing in Rural Uganda and Kenya. Journal of acquired immune deficiency syndromes (1999). 2019 Apr 1;80(4):414–22. doi: 10.1097/QAI.0000000000001939 30807481 PMC6410970

[16] Kenya National Guidelines for Cardiovascular Diseases Management [Internet]. Division of Non-Communicable Diseases, Ministry of Health, Kenya; 2018. Available from: https://www.health.go.ke/wp-content/uploads/2018/06/Cardiovascular-guidelines-2018_A4_Final.pdf

[17] Uganda Clinical Guidelines: National Guidelines for Management of Common Conditions. Republic of Uganda Ministry of Health; 2016.

[18] Van Der Laan M, Rose S. Targeted Learning: Causal Inference for Observational and Experimental Data. New York, NY: Springer; 2011. 628 p. (Springer Series in Statistics).

[19] Balzer LB, van der Laan MJ, Petersen ML, SEARCH Collaboration. Adaptive pre-specification in randomized trials with and without pair-matching. Stat Med. 2016 10;35(25):4528–45. doi: 10.1002/sim.7023 27436797 PMC5084457

[20] Moore KL, van der Laan MJ. Covariate adjustment in randomized trials with binary outcomes: targeted maximum likelihood estimation. Stat Med. 2009 Jan 15;28(1):39–64. doi: 10.1002/sim.3445 18985634 PMC2857590

[21] Rosenblum M, van der Laan MJ. Simple, efficient estimators of treatment effects in randomized trials using generalized linear models to leverage baseline variables. Int J Biostat. 2010 Apr 1;6(1):Article 13. doi: 10.2202/1557-4679.1138 20628636 PMC2898625

[22] Benkeser D, Díaz I, Luedtke A, Segal J, Scharfstein D, Rosenblum M. Improving precision and power in randomized trials for COVID-19 treatments using covariate adjustment, for binary, ordinal, and time-to-event outcomes. Biometrics. 2021 Dec;77(4):1467–81. doi: 10.1111/biom.13377 32978962 PMC7537316

[23] Kwarisiima D, Balzer L, Heller D, Kotwani P, Chamie G, Clark T, et al. Population-Based Assessment of Hypertension Epidemiology and Risk Factors among. PLoS One. 2016;11(5):e0156309. 27232186 10.1371/journal.pone.0156309 PMC4883789

[24] Peiris D, Ghosh A, Manne-Goehler J, Jaacks LM, Theilmann M, Marcus ME, et al. Cardiovascular disease risk profile and management practices in 45 low-income and middle-income countries: A cross-sectional study of nationally representative individual-level survey data. PLoS Med. 2021 Mar;18(3):e1003485. doi: 10.1371/journal.pmed.1003485 33661979 PMC7932723

[25] Havlir DV, Balzer LB, Charlebois ED, Clark TD, Kwarisiima D, Ayieko J, et al. HIV Testing and Treatment with the Use of a Community Health Approach in Rural Africa. New England Journal of Medicine. 2019 Jul 18;381(3):219–29. doi: 10.1056/NEJMoa1809866 31314966 PMC6748325

[26] de Walque D. The use of financial incentives to prevent unhealthy behaviors: A review. Social Science & Medicine. 2020 Sep 1;261:113236. doi: 10.1016/j.socscimed.2020.113236 32781370

[27] Vedanthan R, Kamano JH, Chrysanthopoulou SA, Mugo R, Andama B, Bloomfield GS, et al. Group Medical Visit and Microfinance Intervention for Patients With Diabetes or Hypertension in Kenya. J Am Coll Cardiol. 2021 Apr 27;77(16):2007–18. doi: 10.1016/j.jacc.2021.03.002 33888251 PMC8065205

[28] de Walque D, Chukwuma A, Ayivi-Guedehoussou N, Koshkakaryan M. Invitations, incentives, and conditions: A randomized evaluation of demand-side interventions for health screenings. Soc Sci Med. 2022 Mar;296:114763. doi: 10.1016/j.socscimed.2022.114763 35144225

[29] Choko AT, Corbett EL, Stallard N, Maheswaran H, Lepine A, Johnson CC, et al. HIV self-testing alone or with additional interventions, including financial incentives, and linkage to care or prevention among male partners of antenatal care clinic attendees in Malawi: An adaptive multi-arm, multi-stage cluster randomised trial. PLoS Med. 2019 Jan;16(1):e1002719. doi: 10.1371/journal.pmed.1002719 30601823 PMC6314606

[30] Tanser FC, Kim HY, Mathenjwa T, Shahmanesh M, Seeley J, Matthews P, et al. Home-Based Intervention to Test and Start (HITS): a community-randomized controlled trial to increase HIV testing uptake among men in rural South Africa. J Int AIDS Soc. 2021 Feb;24(2):e25665. doi: 10.1002/jia2.25665 33586911 PMC7883477

[31] Barnabas RV, van Heerden A, McConnell M, Szpiro AA, Krows ML, Schaafsma TT, et al. Lottery incentives have short-term impact on ART initiation among men: results from a randomized pilot study. J Int AIDS Soc. 2020 Jun;23 Suppl 2:e25519. doi: 10.1002/jia2.25519 32589342 PMC7319109

[32] Unger T, Borghi C, Charchar F, Khan NA, Poulter NR, Prabhakaran D, et al. 2020 International Society of Hypertension Global Hypertension Practice Guidelines. Hypertension. 2020 Jun;75(6):1334–57. doi: 10.1161/HYPERTENSIONAHA.120.15026 32370572

[33] Whelton Paul K., Carey Robert M., Aronow Wilbert S., Casey Donald E., Collins Karen J., Cheryl Dennison Himmelfarb, et al. 2017 ACC/AHA/AAPA/ABC/ACPM/AGS/APhA/ASH/ASPC/NMA/PCNA Guideline for the Prevention, Detection, Evaluation, and Management of High Blood Pressure in Adults: A Report of the American College of Cardiology/American Heart Association Task Force on Clinical Practice Guidelines. Hypertension. 2018 Jun 1;71(6):e13–115. DOI: 10.1161/HYP.0000000000000066 29133356

[34] Gebresillassie BM, Debay YB. Characteristics, treatment, and outcome of patients with hypertensive crisis admitted to University of Gondar Specialized Hospital, northwest Ethiopia: A cross-sectional study. J Clin Hypertens (Greenwich). 2020 Dec;22(12):2343–53. 32966697 10.1111/jch.14056 PMC8029926

[35] Mandi DG, Yaméogo RA, Sebgo C, Bamouni J, Naibé DT, Kologo KJ, et al. Hypertensive crises in sub-Saharan Africa: Clinical profile and short-term outcome in the medical emergencies department of a national referral hospital in Burkina Faso. Ann Cardiol Angeiol (Paris). 2019 Oct;68(4):269–74. doi: 10.1016/j.ancard.2019.07.007 31466723

[36] Mchomvu E, Mbunda G, Simon N, Kitila F, Temba Y, Msumba I, et al. Diagnoses made in an Emergency Department in rural sub-Saharan Africa. Swiss Med Wkly. 2019 Jan 28;149:w20018. doi: 10.4414/smw.2019.20018 30715723

[37] Mkoko P, Naidoo S, Niazi M, Tahira A, Godlwana X, Ndesi N, et al. The spectrum, prevalence and in-hospital outcomes of cardiovascular diseases in a South African district hospital: a retrospective study. Cardiovasc J Afr. 2021 Oct 23;32(5):237–42. doi: 10.5830/CVJA-2021-016 34128952 PMC8756043

[38] Nakalema I, Kaddumukasa M, Nakibuuka J, Okello E, Sajatovic M, Katabira E. Prevalence, patterns and factors associated with hypertensive crises in Mulago hospital emergency department; a cross-sectional study. Afr Health Sci. 2019 Mar;19(1):1757–67. doi: 10.4314/ahs.v19i1.52 31149006 PMC6531930

[39] Nkoke C, Noubiap JJ, Dzudie A, M Jingi A, Njume D, Teuwafeu D, et al. Epidemiology of hypertensive crisis in the Buea Regional Hospital, Cameroon. J Clin Hypertens (Greenwich). 2020 Nov;22(11):2105–10. doi: 10.1111/jch.14035 32951311 PMC8029769

[40] Reis KG, Wilson R, Kalokola F, Wajanga B, Lee MH, Safford M, et al. Hypertensive Urgency in Tanzanian Adults: A 1-Year Prospective Study. Am J Hypertens. 2020 Dec 31;33(12):1087–91. doi: 10.1093/ajh/hpaa129 32776154 PMC7947971

[41] Shao PJ, Sawe HR, Murray BL, Mfinanga JA, Mwafongo V, Runyon MS. Profile of patients with hypertensive urgency and emergency presenting to an urban emergency department of a tertiary referral hospital in Tanzania. BMC Cardiovasc Disord. 2018 Aug 2;18(1):158. doi: 10.1186/s12872-018-0895-0 30068315 PMC6090910

