# Supplementary material for: Effect of a one-time financial incentive on linkage to chronic hypertension care in Kenya and Uganda: A randomized controlled trial
Source: PLoS One. 2022 Nov 7;17(11):e0277312. doi: 10.1371/journal.pone.0277312 (PMC9639834; doi:10.1371/journal.pone.0277312)
Supplement: S1 File — (PDF) [file pone.0277312.s001.pdf]

A Multisectoral Strategy to Address Persistent Drivers
of the HIV Epidemic in East Africa (SAPPHIRE)

Sustainable East Africa Research in Community Health (SEARCH) Consortium

Statistical Analysis Plan

Project: Effect of transport voucher and missed visit phone call on linkage to hypertension care in SEARCH SAPPHIRE

Laura B. Balzer, PhD*
Diane V. Havlir, MD**
Maya L. Petersen, PhD***
Matthew D. Hickey, MD**

for the SEARCH Collaboration

December 21, 2021

Version 1.0

*School of Public Health & Health Sciences, University of Massachusetts Amherst, Amherst, USA
**Division of HIV, ID, & Global Medicine, Department of Medicine, UCSF, San Francisco, USA
***School of Public Health, University of California Berkeley, Berkeley, USA

1. Overview 2
2.1 General approach for evaluating intervention effects 3
2.2 Evaluate the effect on linkage to hypertension care at 30 days 4
2.3 Evaluate the effect on linkage to scheduled post-screening appointment 5
2.4 Evaluate the effect on hypertension control at months 3, 6, and 12 5
2.5 Evaluate the effect on retention in hypertension care at 3, 6 and 12 months 6
2.6 Assess individual-level predictors of linkage to care, control, and retention in care 6
Appendix: Power calculations 6
References 7

1. Overview

The SEARCH SAPPHIRE Hypertension Linkage study is an individual randomized controlled trial, designed to test the hypothesis that a transportation voucher and a follow-up phone call will improve linkage to clinic-based hypertension care following community-based screening. The trial takes place in three communities in Kenya and Uganda and began enrollment in June 2021. After community-based screening, individuals diagnosed with hypertension (HTN) were enrolled and given a clinic appointment at the nearest government-run clinic. Participants randomized to intervention (n=100) received a voucher to reimburse transport expenses, redeemable upon clinic linkage at any time following enrollment (even if initial linkage appointment was missed). Intervention participants who missed their initial linkage appointment also received a follow-up phone call to encourage linkage. Control participants (n=100) did not receive a transport voucher or any follow-up if they missed their initial linkage appointment. Upon linkage to care, all participants received integrated, patient-centered hypertension care, detailed elsewhere.[1–3] Treatment guidelines for use of antihypertensive medication were based on standard country guidelines. Additional details of the study procedures are available in the Study Protocol.

The primary objective of this study is to determine whether the intervention improved linkage to HTN care by 30 days. Secondary endpoints include linkage at or before the scheduled appointment, linkage by 3 months, HTN control at follow-up, and retention in hypertension care. We will also characterize changes in HTN severity and evaluate predictors of linkage, HTN control, and retention.

Throughout, the population of interest comprises non-pregnant adults, aged 25 years and older, who do not have a prior diagnosis of hypertension, and who have uncontrolled HTN at baseline as identified through community-based screening (defined as systolic blood pressure ≥140 mmHg or diastolic blood pressure ≥90 mmHg on all three baseline measures). Participants were excluded if they had a blood pressure ≥180/110mmHg and had symptoms of hypertensive emergency – these individuals were immediately transported to the nearest health facility for emergent treatment.

We will provide descriptions of participant flow through the study (i.e., a consort diagram), measurement coverage, and baseline characteristics (e.g., country, age, sex, marital status, occupation, monthly income, education, comorbidities, and baseline severity of hypertension), overall and by arm and sex. We will also report risk factors (N and %) for uncontrolled HTN at baseline, overall and by sex. Additionally, we will report and describe intervention fidelity and delivery of clinical hypertension care. Finally, we will report and describe barriers and facilitators of hypertension care engagement, as assessed through participant self-report at baseline and follow-up.

2.1 General approach for evaluating intervention effects

We will assess the intervention effect with targeted minimum loss-based estimation (TMLE), which provides precision and power gains over an unadjusted analysis by adjusting for baseline predictors of the outcome.[4–7] For a detailed review of TMLE and its relation to other effect estimators in randomized trials, we refer the reader to Colantuoni and Rosenblum.[8] For a recent demonstration of the improved precision offered by TMLE in randomized trials, we refer the reader to Balzer et al. and to Benkser et al..[9,10]

Here, we will use **TMLE with Adaptive Pre-specification**, a fully automated procedure to flexibly adjust for baseline outcome predictors, while maintaining Type-I error control.[11] Specifically, using 10-fold cross-validation, we will chose the optimal approach for estimating the expected outcome given the randomization arm and baseline covariates (a.k.a., the outcome regression) and for estimating the conditional probability of being randomized to the intervention given the baseline covariates (a.k.a., propensity score). Throughout, optimality is defined by using the squared influence curve for the TMLE as loss function. Thereby, we will select the combination of estimators (adjustment variables + approach) of the outcome regression and of the propensity score that minimizes the cross-validated risk estimate and, thus, the cross-validated variance estimate.

Our pre-specified, candidate adjustment variables are age, sex, hypertension severity ("grade 1" as 140-159/90-99 mmHg versus "grade 2 or higher" as ≥160/100 mmHg), site, and nothing (i.e., unadjusted). Our pre-specified, candidate estimators of the outcome regression are main terms, stepwise regression, stepwise regression with all possible pairwise interactions, LASSO, and the mean. Our pre-specified, candidate estimators of the propensity score are main terms, stepwise regression, LASSO, and the mean. In sensitivity analyses, we will also implement that unadjusted effect estimator as the contrasts of average outcomes by arm.

For all endpoints, primary estimates will be for the study sample and on the **relative scale**: $1/n \sum_{i=1}^{n} Y_i(1) \div 1/n \sum_{i=1}^{n} Y_i(0)$, where $Y_i(1)$ denotes the counterfactual outcome for participant *i* under the intervention and $Y_i(0)$ denotes the counterfactual outcome for participant *i* under the control. Secondary comparisons will be on the absolute scale.

For all endpoints, we will test the **null hypothesis** of no improvements in outcomes due to the intervention with a one-sided test at the 5% significance level. We will also report point estimates and 95% confidence intervals for each effect measure and the arm-specific average outcomes. Standard error estimation will be with the estimated influence curve, and statistical inference will follow from the Central Limit Theorem (i.e., using the standard normal distribution).[4]

For all endpoints, we will also examine the intervention effect within **subgroups** defined by sex, age group (<60 years vs. ≥60 years), country, site, baseline HTN severity, and HIV status. In subgroups with fewer than 41 participants, we will limit the candidate estimation approaches to main terms adjustment for a single covariate or the simple mean.

2.2 Evaluate the effect on linkage to hypertension care at 30 days

The primary endpoint is linkage to care by 30 days, evaluated with clinical records. Participants without clinical records indicating linkage to care will be assumed never to have linked. Sensitivity analyses will incorporate self-report, defined as having a clinical record of linkage by 90 days or self-reported linkage to any health facility within the past 3 months. These linkage endpoints are not subject to missingness.

As described above, we will use TMLE with Adaptive Pre-specification to compare linkage to care by 30 days between intervention and control arms and to test the null hypothesis that the intervention did not improve linkage to care by 30 days. Sensitivity analyses incorporating self-report and subgroup analyses will be implemented analogously.

We will also provide descriptive statistics of participants who linked to care by 30 days. Finally, we will generate a graphic of cumulative linkage over time by arm.

2.3 Evaluate the effect on linkage to scheduled post-screening appointment

To assess the effect of the transport voucher, we will again use TMLE to compare linkage to care by the scheduled appointment date between intervention and control arms. We will use the same candidate adjustment variables and approaches as the primary endpoint. This endpoint is also not subject to missingness. We will formally test the null hypothesis that the intervention did not improve linkage to care on or before the scheduled linkage appointment. We will also implement analogous subgroup analyses and provide descriptive statistics of participants who linked to care by their scheduled appointment date.

To elucidate the added value of the transport voucher, we will also compare the intervention effect on linking by 30 days (overall) to the intervention effect on linkage to the scheduled appointment. To do so, we will use the Delta Method to test the null hypothesis that the ratio of relative risks is 1 and that the difference of absolute effects is 0. Here, we will use a two-sided test at the 5% significance level.

2.4 Evaluate the effect on hypertension control at months 3, 6, and 12

Again using TMLE with Adaptive Pre-specification, we will assess the intervention effect on HTN control (all three blood pressure measures <140/90 mmHg) at months 3, 6 and 12 post-enrollment. We will formally test the null hypothesis that the intervention did not improve hypertension control at these timepoints. We will use the same candidate adjustment variables and approaches as the primary endpoint (linkage within 30 days).

In the primary analysis of hypertension control, participants who do not have their blood pressure measured within the endpoint date (e.g., in the window of 2.5-3.5 months post-enrollment for the 3-month endpoint) will be considered to have uncontrolled hypertension. In sensitivity analyses, we will control for incomplete ascertainment of blood pressure measures. These secondary analyses will use TMLE to estimate the intervention effect, while adjusting for differences in characteristics (e.g., arm, sex, age, severity, site) between persons with measurements and persons with missing measurement.

We will again implement subgroup analyses as well as examine control among participants who linked to HTN care within 30 days. We will also evaluate the

intervention impact on HTN severity, defined as participants with grade 2 or higher HTN at follow-up, and on the average of the second and third systolic blood pressure measures at follow-up.

2.5 Evaluate the effect on retention in hypertension care at 3, 6 and 12 months

We will define retention in care as not late for the most recent scheduled hypertension care appointment by 30 days or more. This endpoint is also not subject to missingness. Again, we will use the primary analytic approach of TMLE with Adaptive Pre-specification to assess the intervention effect on retention in hypertension care at 3, 6 and 12 months and to test the null hypothesis that the intervention did not improve retention in care at 3, 6 or 12 months, respectively. For this endpoint, the candidate adjustment variables will be expanded to include baseline HIV status. We will implement analogous subgroup analyses.

2.6 Assess individual-level predictors of linkage to care, control, and retention in care

We will evaluate the following predictors of linkage to care within 30 days: sex, age, country, baseline HTN severity (grade), and baseline HIV status (assessed via testing and self-report). In the primary analysis, TMLE will be used to obtain variable importance measures, capturing the amount of information that a given predictor provides after adjusting for the other predictors. For each of the predictors, we will report the adjusted variables importance measures on the relative scale, treating each baseline predictor in turn as the "exposure" variable, and the rest as the adjustment set. In secondary analyses, we will calculate and report unadjusted (i.e., univariate) associations. We will conduct these analyses overall and stratified by randomized arm. Pooled analyses will include randomization arm as a predictor.

Analogous methods are used to evaluate the predictors of HTN control at 3, 6, and 12 months and retention in care at 3, 6 and 12 months.

Appendix: Power calculations

Sample size and power calculations were based of standard formulas for a two-sample test of proportions and done with *power.prop.test* in *R*.[12] We expect these calculations to be conservative, because of the precision gained through stratified randomization, through covariate adjustment during the analysis, and through our use of a one-sided hypothesis test.

We estimated 100 participants/arm would provide 80% power to detect at least a 19.3% absolute increase in linkage within 30 days from 50% under the control. As shown in the following Figure, even with 25% fewer participants enrolled (from 100 to 75 participants/arm) and lower or higher linkage under the standard-of-care, these calculations suggest we would be well-powered to detect at least a 22.7% absolute increase in linkage.

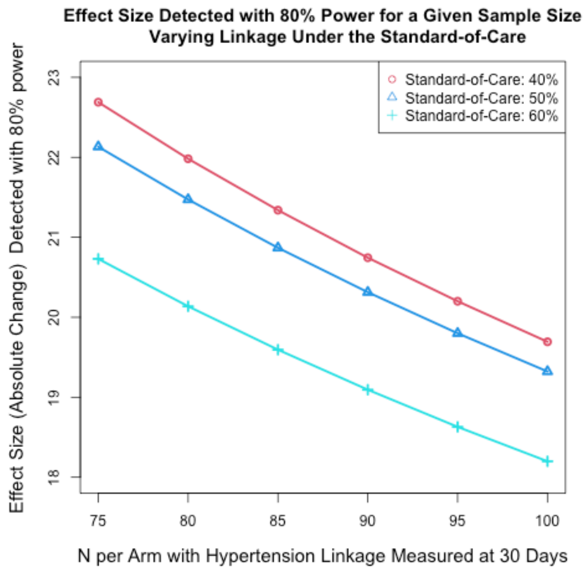

References

1. Kwarisiima D, Atukunda M, Owaraganise A, et al. Hypertension control in integrated HIV and chronic disease clinics in Uganda in the SEARCH study. *BMC Public Health*. 2019;19(1):511. doi:10.1186/s12889-019-6838-6

2. Kwarisiima D, Kamya M, Owaraganise A, et al. High rates of viral suppression in adults and children with high CD4+ counts using a streamlined ART delivery model in the SEARCH trial in rural Uganda and Kenya. *J Int AIDS Soc*. 2017;Jul 21(20).

3. Havlir DV, Balzer LB, Charlebois ED, et al. HIV Testing and Treatment with the Use of a Community Health Approach in Rural Africa. *N Engl J Med*. 2019;381(3):219-229.

4. van der Laan M, Rose S. *Targeted Learning: Causal Inference for Observational and Experimental Data*. Springer; 2011.

5. Moore KL, van der Laan MJ. Covariate Adjustment in Randomized Trials with Binary Outcomes: Targeted Maximum Likelihood Estimation. *Stat Med*. 2009;28(1):39-64. doi:10.1002/sim.3445

6. Rosenblum M, van der Laan MJ. Simple, Efficient Estimators of Treatment Effects in Randomized Trials Using Generalized Linear Models to Leverage Baseline Variables. *Int J Biostat*. 2010;6(1):Article 13. doi:10.2202/1557-4679.1138

7. van der Laan MJ, Rose S. *Targeted Learning in Data Science*. Springer; 2018.

8. Colantuoni E, Rosenblum M. Leveraging Prognostic Baseline Variables to Gain Precision in Randomized Trials. *Stat Med*. 2015;34(18):2602-2617.

9. Balzer LB, van der Laan M, Ayieko J, et al. Two-Stage TMLE to Reduce Bias and Improve Efficiency in Cluster Randomized Trials. *Biostatistics*. 2021;kxab043. https://doi.org/10.1093/biostatistics/kxab043

10. Benkeser D, Díaz I, Luedtke A, Segal J, Scharfstein D, Rosenblum M. Improving precision and power in randomized trials for COVID-19 treatments using covariate adjustment, for binary, ordinal, and time-to-event outcomes. *Biometrics*. 2021;n/a(n/a):1-15. doi:10.1111/biom.13377

11. Balzer L, van der Laan MJ, Petersen M, SEARCH Collaboration. Adaptive Pre-specification in Randomized Trials With and Without Pair-Matching. *Stat Med*. 2016;35(10):4528-4545. doi:10.1002/sim.7023

12. R Core Team. *R: A Language and Environment for Statistical Computing*. R Foundation for Statistical Computing; 2020. http://www.R-project.org
