# Supplementary material for: Effect of a one-time financial incentive on linkage to chronic hypertension care in Kenya and Uganda: A randomized controlled trial
Source: PLoS One. 2022 Nov 7;17(11):e0277312. doi: 10.1371/journal.pone.0277312 (PMC9639834; doi:10.1371/journal.pone.0277312)
Supplement: S3 File — (DOCX) [file pone.0277312.s003.docx]

**A Multisectoral Strategy to Address Persistent Drivers of the HIV Epidemic in East Africa (SAPPHIRE): Hypertension Linkage Study Protocol**

Sustainable East Africa Research in Community Health (SEARCH) Consortium

Clinical Trial Phase: IV

**Funded by: National Institutes of Health**

**Protocol Chair: Diane Havlir, MD**

**Protocol Co-Chairs: Moses Kamya, MBChB, MPH, PhD**

**Maya Petersen, MD, PhD**

**Protocol Vice-Chair: Gabriel Chamie, MD, MPH**

Version Date: 8 March 2022

**TABLE OF CONTENTS**

[1. KEY ROLES 4](#_Toc104285901)

[2. LIST OF ABBREVIATIONS 6](#_Toc104285902)

[3. PROTOCOL SUMMARY 7](#_Toc104285903)

[4. INTRODUCTION 8](#_Toc104285904)

[4.1. Background 8](#_Toc104285905)

[4.2. Study Hypothesis for Primary Objective 8](#_Toc104285906)

[5. OBJECTIVES 8](#_Toc104285907)

[5.1. Primary Objective 8](#_Toc104285908)

[6. STUDY DESIGN 8](#_Toc104285909)

[7. STUDY POPULATION 9](#_Toc104285910)

[7.1. Community-Level Inclusion Criteria 9](#_Toc104285911)

[7.1.1. Community Level Inclusion Criteria 9](#_Toc104285912)

[7.1.2. Community Level Exclusion Criteria 9](#_Toc104285913)

[7.2. Individual Level Inclusion Criteria 9](#_Toc104285914)

[7.3. Recruitment Process 9](#_Toc104285915)

[8. INTERVENTIONS 10](#_Toc104285916)

[9. STUDY PROCEDURES/EVALUATIONS 10](#_Toc104285917)

[9.1. Randomization 10](#_Toc104285918)

[9.2. Study Procedures 10](#_Toc104285919)

[9.2.1. Clinician Training 10](#_Toc104285920)

[9.2.2. Informed Consent and Enrollment 10](#_Toc104285921)

[9.2.3. Hypertension Intervention Components 10](#_Toc104285922)

[9.2.4. Hypertension Linkage to Care and Treatment 11](#_Toc104285923)

[9.3. Measurements 11](#_Toc104285924)

[9.4. Participant Discontinuation 12](#_Toc104285925)

[10. ASSESSMENT OF SAFETY 13](#_Toc104285926)

[10.1. Safety Assessment Overview 13](#_Toc104285927)

[10.2. Adverse Event Procedures and Reporting Requirements 13](#_Toc104285928)

[11. DATA HANDLING AND RECORDKEEPING 14](#_Toc104285929)

[11.1. Data Management Responsibilities 14](#_Toc104285930)

[11.2. Essential/Source Documents and Access to Source Data/Documents 14](#_Toc104285931)

[11.3. Quality Control and Quality Assurance 14](#_Toc104285932)

[12. CLINICAL SITE MONITORING 15](#_Toc104285933)

[13. ADMINISTRATIVE PROCEDURES 15](#_Toc104285934)

[13.1. Regulatory Oversight 15](#_Toc104285935)

[13.2. Study Implementation 15](#_Toc104285936)

[13.3. ClinicalTrials.gov 15](#_Toc104285937)

[14. HUMAN SUBJECTS PROTECTIONS 16](#_Toc104285938)

[14.1. Institutional Review Board/Ethics Committee 16](#_Toc104285939)

[14.2. Vulnerable Participants 16](#_Toc104285940)

[14.2.1. Pregnant Women and Fetuses 16](#_Toc104285941)

[14.2.2. Children 16](#_Toc104285942)

[14.2.3. Illiterate Participants 16](#_Toc104285943)

[14.3. Informed Consent 16](#_Toc104285944)

[14.3.1. Informed Consent Process 16](#_Toc104285945)

[14.3.2. Documentation of Informed Consent 17](#_Toc104285946)

[14.3.3. Waiver of Informed Consent 17](#_Toc104285947)

[14.4. Risks 18](#_Toc104285948)

[14.5. Social Impact Events 18](#_Toc104285949)

[14.6. Benefits 18](#_Toc104285950)

[14.7. Compensation 19](#_Toc104285951)

[14.8. Participant Privacy and Confidentiality 19](#_Toc104285952)

[14.9. Study Discontinuation 19](#_Toc104285953)

[14.10. Community Advisory Board and Other Relevant Stakeholders 19](#_Toc104285954)

[15. STATISTICAL ANALYSIS 21](#_Toc104285955)

[15.1. Overview 21](#_Toc104285956)

[15.2 Individually randomized trial procedures 21](#_Toc104285957)

[15.2.1 Power calculations 21](#_Toc104285958)

[16. PUBLICATION POLICY 22](#_Toc104285959)

[17. REFERENCES 22](#_Toc104285960)

# 1. KEY ROLES

**Diane Havlir, MD**

Chair, University of California, San Francisco

Address: UCSF Box 0874, San Francisco, CA 94143-0874, U.S.A.

Phone Number: +1-415-476-4082 ext. 424

Email: [diane.havlir@ucsf.edu](mailto:diane.havlir@ucsf.edu)

**Moses R. Kamya, MBChB, MMed, PhD**

Co-Chair, Makerere University

Address: Department of Medicine, P.O. Box 7072, Kampala, Uganda

Phone Number: +256-414-533200

Email: [mkamya@infocom.co.ug](mailto:mkamya@infocom.co.ug)

**Maya L. Petersen, MD, PhD**

Co-Chair, University of California, Berkeley

Address: School of Public Health, 5402 Berkeley Way West, Berkeley, CA 94720-7358, U.S.A.

Phone Number: +1-510-642-0563

**Gabriel Chamie, MD, MPH**

Vice-Chair, University of California, San Francisco

Address: UCSF Box 0874, San Francisco, CA 94143-0874, U.S.A.

Phone Number: +1-415-476-4082 ext. 445

Email: [gabriel.chamie@ucsf.edu](mailto:gabriel.chamie@ucsf.edu)

**Laura Balzer, PhD**

Investigator and Protocol Statistician, University of Massachusetts, Amherst

Address: School of Public Health and Health Sciences, Amherst, MA 01003-9304, U.S.A.

Phone Number: +1-413-545-9464

Email: [lbalzer@umass.edu](mailto:lbalzer@umass.edu)

**Jane Kabami, MPH**

Investigator, Infectious Diseases Research Collaboration

Address: P.O. Box 7475, Kampala, Uganda

Phone Number: +256-706-315810

Email: [jkabami@idrc-uganda.org](mailto:jkabami@idrc-uganda.org)

**Elijah Kakande, MBChB**

Investigator, Infectious Diseases Research Collaboration

Address: P.O. Box 4745, Kampala, Uganda

Phone Number: +256-779-386616

Email: [rkakande@idrc-uganda.org](mailto:rkakande@idrc-uganda.org)

**Asiphas Owaraganise, MBChB**

Investigator, Infectious Diseases Research Collaboration

Address: P.O. Box 4745, Kampala, Uganda

Email: asiphas@gmail.com

**James Ayieko, MBChB, MPH, PhD**

Investigator, Adult and Adolescent Studies, Kenya Medical Research Institute

Address: P.O. Box 517-20107, Njoro, Kenya

Phone Number: +254-720-925262

Email: [jimayieko@gmail.com](mailto:jimayieko@gmail.com)

**Norton Sang, MA**

Investigator, Kenya Medical Research Institute

Address: P.O. Box 35-4132, Mega City, Kisumu, Kenya

Phone Number: +254-715-778930

Email: [nortonsang@gmail.com](mailto:nortonsang@gmail.com)

**Matthew Hickey, MD**

Investigator, University of California, San Francisco

Address: UCSF Box 0874, San Francisco, CA 94143-0874, U.S.A.

Phone Number: +1-415-476-4082

Email: matt.hickey@ucsf.edu

# 2. LIST OF ABBREVIATIONS

**CHR**: Committee on Human Research

**CHV**: Community health volunteer

**CRF**: Case report form

**CVD**: Cardiovascular disease

**DALYs**: Disability-adjusted life years

**DM**: Diabetes mellitus

**DSMB**: Date Safety Monitoring Board

**FGD**: Focus group discussion

**HIV**: Human immunodeficiency virus

**HTN**: Hypertension

**IDI**: In-depth interview

**IPV**: Intimate partner violence

**IRB**: Institutional Review Board

**KEMRI**: Kenya Medical Research Institute

**MoH**: Ministry of Health

**NASCOP**: National AIDS and STI Control Programme

**PRECEDE**: Predisposing, Reinforcing, and Enabling Constructs in Educational Diagnosis and Evaluation

**SAP**: Statistical analysis plan

**RE-AIM**: Reach, Effectiveness, Adoption, Implementation and Maintenance

**SEARCH**: Sustainable East Africa Research in Community Health

**SOMREC**: Makerere University School of Medicine - Research and Ethics Committee

**SOPs**: Standard Operating Procedures

**SSA**: Sub-Saharan Africa

**TDF/FTC**: Tenofovir disoproxil fumarate/Emtricitabine

**TMLE**: Targeted maximum likelihood estimation

**UCSF**: University of California, San Francisco

**UNCST**: Uganda National Council for Science and Technology

# 3. PROTOCOL SUMMARY

**Title:** A Multisectoral Strategy to Address Persistent Drivers of the HIV Epidemic in East Africa (SAPPHIRE): Hypertension Linkage Study

**Sample Size:** 200 participants ≥25 years of age

**Participating Sites:** 3 communities in rural western Kenya and rural western Uganda, that: a) are geopolitical units (i.e. parish in Uganda, or sub-location in Kenya) of ~10,000 persons each (5,000 ≥15 years) within the catchment area of government health clinics; b) have HIV prevalence similar to the region using Ministry of Health data; c) have a letter of commitment to the study from the local political leader; and d) are of sufficient distance from each other to avoid contamination.

**Hypothesis:** Transport vouchers and phone call follow-up for missed visits will improve linkage to hypertension care following community-based screening.

**Study Population:**

- Inclusion criteria:
  - Age ≥25 years
  - Blood pressure ≥140/90 mmHg on three repeated measures during community-based hypertension screening
  - Resident (by self-report) within catchement area of referral health center
- Exclusion criteria:
  - Plan to out-migrate from the catchment area of referral health center within 30 days of screening visit
  - Already engaged in hypertensive care (by self-report)
  - Blood pressure measure of ≥180/110 mmHg during screening and symptoms of hypertensive emergency
  - Pregnant

**Primary Objective:** Assess the effect of the intervention on hypertension linkage and control

**Study Design:** Individually randomized trial (blocked and stratified on community and sex) with 1:1 randomization to linkage to care intervention or standard referral to clinic-based hypertension care.

**Interventions:** Study interventions include a one-time transport voucher reimbursable upon linkage to hypertension care (value ~$5 USD) and a follow-up phone call if not linked to care within 7 days.

**Study Endpoints:**

- **Primary endpoint:** linkage to care by 30 days
- **Secondary endpoint:** Hypertension control (blood pressure <140/90 mmHg) at 90 days

**Study Duration:** 12 months.

**Sample Size and Power:** We will enroll up to 200 participants (100/arm) based on assuming 50% linkage in the non-intervention group for 80% power to detect a 20% or greater increase in linkage.

# 4. INTRODUCTION

## 4.1. Background

**The burden of CVD-associated morbidity and mortality is rapidly rising across sub-Saharan Africa (SSA).**^1,2^ Over the next two decades, an estimated 34 million excess years of life will be lost across SSA if action is not taken to address key risk factors for CVD.^3^ Hypertension is the most important risk factor for CVD and is highly prevalent in SSA; at least 35% of adults aged 40-60 years have hypertension.^4–6^ CVD risk is further increased among people living with HIV (PLH), and the burden of CVD is expected to increase among PLH as this population ages.^7–13^ To address the rising burden of CVD in both PLH and HIV-seronegative populations, there is a critical need for strategies to improve hypertension linkage, treatment initiation, and long-term retention in care across SSA.

**There is strong evidence from randomized controlled trials that antihypertensive medications reduce morbidity and mortality from CVD among people with hypertension; however, hypertension diagnosis and treatment is limited in SSA.**^14–17^ Antihypertensive medications are inexpensive and treatment is highly cost-effective in low-resource settings, including Kenya and Uganda.^18–21^ However, numerous system and patient-level factors act as barriers to sustained benefit from treatment.^22^ Across SSA, an estimated 27% of those with hypertension are diagnosed, 18% are currently on treatment, and only 7% have controlled hypertension.^5^ Limitations in diagnosis and treatment are similar in East Africa,^2,23,24^ even among those at greatest CVD risk.^25^

**Strategies to improve community screening and linkage to care are critical first steps in improving hypertension treatment.** Evidence from the SEARCH study demonstrates that high levels of hypertension awareness can be achieved through multi-disease community health campaigns.^26^ However, linkage to care following community screening was less than 50% in the SEARCH study^27^ and in other studies evaluating interventions to improve hypertension screening and linkage to care.^28,29^ Long distances to clinic, high transportation costs, and other costs associated with hypertension care contribute to low levels of linkage to hypertension care.^22,30,31^ Our group previously showed improvements in linakge to HIV care using transport vouchers designed to offset costs associated with linkage to care.^32^ In this study, we test the hypothesis that transport voucehrs will improve linkage to hypertension care following community-based screening.

## 4.2. Study Hypothesis for Primary Objective

Transport vouchers and phone call follow-up for missed visits will improve linkage to hypertension care following community-based screening.

# 5. OBJECTIVES

## 5.1. Primary Objective

Assess the effect of the intervention on hypertension linkage and control.

# 6. STUDY DESIGN

This is an individually randomized trial designed to evaluate to assess effectiveness, fidelity and cost of an intervention to improve linkage to hypertension care following community-based screening.

# 7. STUDY POPULATION

## 7.1. Community-Level Inclusion Criteria

The study will take place in 2 rural communities in Kenya and 1 rural community in Uganda.

### 7.1.1. Community Level Inclusion Criteria

1. Non-adjacent geopolitical units in western Uganda and western Kenya.

1. Most recent census population between 9,000 and 11,000 individuals.
2. Served by an ART/PrEP/PEP providing health center with antenatal and family planning services.
3. Community leader commitment for study participation and implementation.

### 7.1.2. Community Level Exclusion Criteria

1. Presence of ongoing community-based interventions that provide HIV prevention or treatment outside of the current in-country treatment guidelines.

1. An urban setting defined as a city with a population of 100,000 or more inhabitants.
2. Absence of a health center able to provide ART and hypertension treatment.

## 7.2. Individual Level Inclusion Criteria

**Table 1. Inclusion/Exclusion Criteria**

| **Inclusion Criteria** | **Exclusion Criteria** |
| --- | --- |
| Hypertension Linkage and Treatment Interventions   1. Age ≥25 years 2. Blood pressure ≥140/90 mmHg on three repeated measurements during community-based hypertension screening 3. Resident (by self-report) within catchment area of referral health center | Hypertension Linkage and Treatment Interventions  1. <25 years  2. Plan to out-migrate from the catchment area of referral health center within 30 days of screening visit  3. Already engaged in hypertensive care (by self-report)  4. Blood pressure measure of ≥180/110 mmHg during screening and symptoms of hypertensive emergency  5. Pregnant |

## 7.3. Recruitment Process

The study will examine an intervention to promote linkage to hypertension care among persons (age ≥25), regardless of HIV status, who have elevated blood pressure (≥140/90 mmHg) identified during community-based hypertension screening. Study leadership will meet with clinic directors in the communities to explain the study purpose and goals. In collaboration with study clinic leadership, study staff will approach consecutive potential participants for participation in the study.

# 8. **INTERVENTIONS**

The study intervention will entail a one-time transport voucher redeemable upon linkage to clinical hypertension care at the community government-run health facility. The value of the voucher will be approximately ~$5 USD and not more than $10 USD. Intervention participants will additionally receive up to three follow-up phone calls in the event of missed initial linkage visits.

# 9. STUDY PROCEDURES/EVALUATIONS

## 9.1. Randomization

We will randomize individuals to the active treatment intervention or to standard-of-care as shown below.

**Table 2: Randomization**

| **Intervention** | **Total number of participants** |
| --- | --- |
| Hypertension Linkage - Individual randomization linkage to care | 200 |

After screening for eligibility, participants will be randomized at the time of enrollment via computer-generated random assignment.

## 9.2. Study Procedures

### 9.2.1. Clinician Training

Prior to recruitment and enrollment of participants, staff providing hypertension treatment will receive training that will include, but will not be limited to, the following components:

- Country hypertension diagnosis and treatment guidelines
- Patient-centered care including friendly services and integrated hypertension care provided with HIV care

Possible side effects of commonly prescribed hypertension medications, tailored to regimens recommended by country MoH guidelines, will be reviewed with staff, along with counseling and support mechanisms to encourage adherence as appropriate

### 9.2.2. Informed Consent and Enrollment

Prior to consent, participants will be screened to determine eligibility in the intervention. A waiver of consent will be obtained to confirm eligibility during the screening process. Written informed consent to participate in the study will be obtained from all participants. Consent forms will be translated from the original English to the language(s) spoken in the community. The consent form will be read to participants in their local language. Participants who agree to take part and sign the consent form will be enrolled in the study.

### 9.2.3. Hypertension Intervention Components

The study intervention promotes linkage to care among persons screening positive for hypertension during community-based screening, and provision of care in a multi-disease chronic care model that includes service for HIV, hypertension, and diabetes. ART will be delivered and monitored according to country guidelines and not provided by the study. Country guidelines for unsupressed viral loads will be followed. Diabetes and hypertension care will be provided using MoH standards. Table 3 describes the Dynamic Treatment intervention components.

**Table 3. Dynamic Treatment Intervention Components**

| **Component** | **Description** |
| --- | --- |
| Hypertension Linkage intervention | 1. Travel voucher (financial incentive) conditional on linkage to hypertensive care |

### 9.2.4. Hypertension Linkage to Care and Treatment

**Linkage to care:** Following community mobilization, study staff will offer community-based screening for hypertension to persons ≥25 years old during mobile outreach to community venues. Persons screened who have blood pressure measurements ≥140/90 mmHg after three repeated measurements at a single community-based screening will be eligible for enrollment into the hypertension linkage to care trial. Eligible adults who consent to participate will then choose a scratch card that reveals whether they have been randomized to receive a transport voucher that is reimbursed upon linkage to hypertension care (intervention) or randomized to read a message thanking them for screening and encouraging them to link to care for their hypertension clinic appointment (control). All participants will be provided information about hypertension and where to access hypertension care and scheduled for a clinic appointment within 14 days of screening/enrollment. Participants with blood pressure ≥160/100 will be scheduled for a clinic appointment within 3 days of screening/enrollment.

Study staff will provide transport reimbursement (up to $10 USD) to Hypertension Linkage trial intervention participants who link to care within 30 days of screening.

**Clinic Services:** All community members who screen positive for hypertension during community-based screening and link to the local government-run clinic (regardless of participation in the trial) will be offered repeat measurement of blood pressure, enrollment into the hypertension clinic, and evaluation for anti-hypertensive treatment.

## 9.3. Measurements

The primary outcome for the Hypertensive Linkage Intervention will be linkage to hypertension care at the local government clinic within 30 days of screening positive for high blood pressure during community screening. Secondary outcomes for the Hypertension Linkage Intervention trial will include linking community members to hypertension care on the date of their scheduled appointment and blood pressure control (defined as systolic blood pressure <140 mmHg and diastolic blood pressure <90 mmHg) at 3-months post-enrollment.

Process and implementation measures for the Dynamic Treatment measures will be obtained from clinic-based records and logs (Table 4).

**Table 4. Phase A Dynamic Treatment Process Measures**

| **Setting** | **Intervention Fidelity** | **Patient-level Intervention Process Outcomes** |
| --- | --- | --- |
| Hypertension  Linkage Intervention | Proportion of intervention participants who receive transport reimbursement upon linkage to care | Linkage to scheduled appointment |

## 9.4. Participant Discontinuation

Participants are free to withdraw from active participation in the study at any time upon request. An investigator may discontinue a participant from the study for the following reasons:

- The participant meets a previously unrecognized exclusion criterion
- Significant non-compliance with the study
- Any event or situation that occurs such that participation would not be in the best interest of the participant

# 10. ASSESSMENT OF SAFETY

## 10.1. Safety Assessment Overview

All medications provided in this study are standard care for the intervention described. Hypertension treatment will be provided according to country MoH guidelines. HIV testing and care will be performed according to MoH guidelines, including regular safety assessments such as VL for HIV-positive individuals in care, and other standard measurements.

Care will be taken to protect the privacy of participants in this study. However, there is a risk that others may inadvertently see participants’ research or medical information, and thus privacy may be compromised.

## 10.2. Adverse Event Procedures and Reporting Requirements

As all intervention procedures in this study are standard care and prevention procedures recommended by the Uganda and Kenya MoH, and risks to participation in general are minimal, this study will not monitor or collect data on all adverse events. In the unlikely event that a serious adverse event (SAE) is considered possibly, probably or definitely related to the study, or in the event of unexpected incidents or protocol violations, reporting to University of California-San Francisco CHR, Makerere University SOMREC, and Kenya Medical Research Institute SERU IRBs will be done as outlined in Table 5. Investigators will be monitoring events in the intervention arms, and suspected events will be reviewed by study investigators prior to submission to the IRB.

**Table 5. Event Reporting Timeline**

| **Institution** | **Type of Events** | **When to Report** |
| --- | --- | --- |
| **UCSF-Committee on Human Subjects Research (CHR)** | - External (off-site) adverse event that UCSF PI determines changes the study risks or benefits, OR necessitates modification to the CHR-approved consent document(s) and/or the CHR-approved application/ protocol - On-site protocol violations (events occurring at UCSF) | - Within 10-working days of PI’s awareness |
| **Makerere University**  **School of Medicine Research Ethics Council (SOMREC)** | - All Serious or Unexpected events considered possibly, probably or definitely related - All hospitalizations except for those related to routine child-bearing admissions | - Fatal or life-threatening events within 3 working days of awareness - All other SAEs within 7 calendar days |
| **KEMRI Scientific and Ethics Review Unit (SERU)** | - All Serious or Unexpected events considered possibly, probably or definitely related - All hospitalizations | - Study related events within 24 hours of awareness - Unrelated events within 10 working days of awareness |
| **National Institute of Allergy and Infectious Diseases (NIAID)** | - All Serious or Unexpected events considered possibly, probably or definitely related | - At the time of reporting to IRBs |

# 11. DATA HANDLING AND RECORDKEEPING

## 11.1. Data Management Responsibilities

Data management for this study will be overseen by the Infectious Diseases Research Collaboration (IDRC) Data Management Center located in the main IDRC research complex in Nakasero, Kampala, Uganda. The Data Management Center (DMC) is also responsible for providing IT support to all staff members.

**Data Management System:** For any study components using paper data collection, IDRC has designed and developed a custom Data Management System that is used to manage the data, using Microsoft SQL Server as the backend for all data storage. MS Access is used for double data entry and SAS programs are used for comparing 1st and 2nd entry, and generating discrepancy reports. In addition, the DMC has developed custom Visual Basic programming for direct survey and data entry in the field. Data Transformation Services/ SQL Server Integration Services (DTS/SSIS) packages are used to automatically import/export any new data; and stored procedures written in T-SQL are used to automatically generate new data queries on a daily basis. There is a web interface to the whole system written in ASP.NET to allow users to view the data, view reports, and modify the data they are authorized to access. With regular backups and a full audit trail, the whole system is regulatory compliant (21 CFR Part 11).

## 11.2. Essential/Source Documents and Access to Source Data/Documents

Data is collected as a mixture of paper Case Report Forms (CRFs) and logs, and electronic data captured from health facility databases and field data tablets. Data is entered locally and uploaded to the DMC after quality assurance measures have taken place. Only study investigators and staff affiliated with the study will have access to source data.

External data sharing will be made through a data request proposal procedure coordinated by the PIs and the IDRC Data Department. A standardized data request sheet will be completed by outside investigators and reviewed by the senior management and/or the Principal Investigator. Data and associated documentation will be available to users under a data-sharing agreement that provides for: (1) a commitment to using the data only for research purposes and not to identify any individual participant; (2) a commitment to securing the data using appropriate computer technology; and (3) a commitment to destroying or returning the data after analyses are completed. User registration is required to access or download files. As part of the registration process, users must agree to the conditions of use governing access to the public release data, including restrictions against attempting to identify study participants, destruction of the data after analyses are completed, reporting responsibilities, restrictions on redistribution of the data to third parties, and proper acknowledgement of the data resource.

## 11.3. Quality Control and Quality Assurance

The data management system is designed to collect, transfer, and store data for Data Management Center studies. Data from any paper records will be 100% double-entered into the system via Microsoft Access Databases or Web-based data entry screens. Locally collected data is compressed, password-protected, and then securely uploaded to a cloud based server (Network Solutions, Inc.) using secure-FTP via the FileZilla application. Cloud-based uploads are downloaded daily to the Kampala DMC. After the data has been input into the Data Management Center server, it is electronically verified and is then written to the SQL database. Edit checks or queries are run nightly and the results are posted to a secure clinical trials website so that the sites can electronically address problems with the data the next day. The site corrects data via the website and the database is updated automatically.

In order to ensure data security and integrity, the following measures will be implemented:

- All members of the study team will be educated in the study protocol prior to the onset of the study.
- Detailed Standard Operating Procedures (SOPs) will be written for all project activities and be provided to relevant team members.
- Team members will be thoroughly trained on the SOPs.
- Where applicable, team members will receive additional training on the use of tablet computers.
- All data transcribed from paper will be double data entered or verified.
- All electronic data will be backed up regularly.
- All data will be transferred to the main Data Center in Kampala to the secure server. This sever is backed up on a daily basis and a monthly backup is stored off-site.
- All computers, including the tablets, will be password protected.
- All computers, including tablets, will be locked in a secure room each night.
- Any Log Books and CRF’s will be locked in a secure room each night.

# 12. CLINICAL SITE MONITORING

Site monitors under contract to the University of California, San Francisco will visit participating clinical research sites to review participants records, including consent forms, CRFs, and laboratory records to ensure protection of study participants, compliance with IRB-approved protocol/amendments, and accuracy and completeness of records. The monitors will inspect sites’ regulatory files to ensure that local regulatory requirements, in addition to U.S. Federal regulations, are being followed.

# 13. ADMINISTRATIVE PROCEDURES

## 13.1. Regulatory Oversight

The proposed research study will be reviewed and approved by the IRBs of all the participating institutions in the U.S., Uganda and Kenya. This includes the UCSF Committee on Human Research (CHR), the Makerere University School of Medicine - Research and Ethics Committee (SOMREC), the Uganda National Council of Science and Technology (UNCST), and the Kenya Medical Research Institute (KEMRI). As described in Section 15.1., this study will employ four levels of data and participant safety monitoring including Study Steering Committee oversight, US and host country IRB review, Data Safety Monitoring Board (DSMB) Review, and External Clinical Site Monitoring. Section 10.2 describes event reporting guidelines to individual IRBs overseeing this study.

## 13.2. Study Implementation

No study activities will commence prior to approval of US and host country IRBs and all other required local and international institutions. This study is funded and supported by the National Institutes of Health.

## 13.3. ClinicalTrials.gov

This protocol will be registered in ClinicalTrials.gov.

# 14. HUMAN SUBJECTS PROTECTIONS

## 14.1. Institutional Review Board/Ethics Committee

The proposed research study will be reviewed and approved by the IRBs of all the participating institutions in the U.S., Uganda and Kenya. This includes the UCSF Committee on Human Research (CHR), the Makerere University School of Medicine - Research and Ethics Committee (SOMREC), the Uganda National Council of Science and Technology (UNCST), and the Kenya Medical Research Institute (KEMRI).

## 14.2. Vulnerable Participants

Many persons living in these regions of East Africa are economically or educationally disadvantaged, by American or African standards. The investigative team believes that the opportunity to participate in this study should be made available to people independent of economic and educational attainment. Participants in rural populations and those who are educationally disadvantaged are among those most at risk in their country for poor HIV care outcomes, and it is important to provide opportunities to participate in research to all people independent of their literacy. If a potential study participant is unable to read or write, his or her fingerprint will substitute for a signature, and a signature from a witness to the informed consent procedures will be obtained. Participants taking part in activities for which a verbal consent is required will have the consent text read to them in their language. All tests and treatments provided are standard of care. Prisoners (incarcerated persons) will not be included in the research during incarceration.

### 14.2.1. Pregnant Women and Fetuses

Pregnant women will not take part in the study. Pregnant women with hypertension identified through community-based screening will be immediately transported to the antenatal clinic at the nearest government health facility for additional evaluation and treatment.

### 14.2.2. Children

Children will not take part in the study due to the different evaluation and care models required for children with hypertension.

### 14.2.3. Illiterate Participants

The study will enroll participants who may be economically or educationally disadvantaged. Participants in rural populations and those who are educationally disadvantaged are among those most at risk in their country for poor HIV care outcomes, and it is important to provide opportunities to participate in research to all people independent of their literacy. If a potential study participant is unable to read or write, his or her fingerprint will substitute for a signature, and a signature from a witness to the informed consent procedures will be obtained. Participants taking part in activities for which a verbal consent is required will have the consent text read to them in their language. As with other groups, illiterate participants will be provided sufficient time to be read the consent form or ask questions in order to understand study procedures.

## 14.3. Informed Consent

### 14.3.1. Informed Consent Process

All written and verbal consent forms will be translated into the local language and back translated into English to ensure correct use of language. Consent forms will be read aloud to participants by trained staff. The informed consent will describe the purpose of the study, all the procedures involved, and the risks and benefits of participation. Interviewers will ask participants to summarize the study and explain the reasons why they want to participate. Either a signature or a thumbprint (for those who cannot read) will be acceptable to confirm informed consent for participation in the study, in the case of written consent forms. Witnesses independent from the study will be required to be present for consent discussion and co-sign consent forms for participants who are illiterate. Affirmation of verbal consent will be obtained through recording responses on verbal consent logs. Consent documentation may be tablet instead of paper-based to maintain optimal infection control.

To minimize the likelihood of persons feeling pressured to participate in research we will emphasize the concepts of individual voluntary choice and the need for research assistants to respect the voluntary choice of others during the process of obtaining informed consent. Study participants will also be informed that participation can be stopped at any point during the study at their request. Descriptions of individual consent forms and procedures are below.

**Written Consent:** Participants taking part in the Hypertension Linkage Trial, will meet with study staff to review the services offered. After being given adequate time to review the consent form and have all questions answered, participants then provide written informed consent to take part in the trial.

**Verbal Consent for Hypertension Screening:** Participants taking part in screening for the hypertension linkage or treatment studies will meet with study staff to review the services offered and provide informed verbal consent to take part. The verbal consent form will be read to them in the local language and confirmation of consent will be documented as affirmation of their agreement to participate.

**Table 6. Consent Populations and Procedures**

| **Consent** | **Number of participants** | **Data Collection Tools** |
| --- | --- | --- |
| Verbal consent for Hypertension screening; Written consent for Hypertension Linkage RCT enrollment | Hypertension linkage: up to 250 (control and intervention) participants | CRFs, MoH care records and logs, surveys |

### 14.3.2. Documentation of Informed Consent

Documentation of written informed consent and verbal consent will be recorded on informed consent logs for all study activities. The logs will contain the participant’s study ID, clinic ID, initials, name, language of consent used, and whether the individual signed the document or provided a fingerprint in the case of written consents, or verbally affirmed their consent in the case of verbal consents.

### 14.3.3. Waiver of Informed Consent

A waiver of consent will be obtained from all IRBs for the collection of data from medical records related to HIV, prevention, and other disease care records—as shown in Table 6. The waiver of consent will meet the following criteria: (1) the research involves no more than minimal risk to the subjects; (2) the waiver of consent will not adversely affect the rights and welfare of the subjects; and (3) the research could not practicably be carried out without the waiver.

## 14.4. Risks

**Privacy:** The primary risk to study participation is breach of privacy. Care will be taken to protect the privacy of participants and parents/guardians, as described in Section 14.8. However, there is a risk that others may inadvertently see patients’ medical information, and thus their privacy may be compromised. Further details on the social impact of such potential privacy breaches are described in section 14.5.

**Psychological Discomfort:** There is the possibility of some psychological discomfort that may arise from questions asked about other medical conditions during surveys. We will train all study staff involved with surveys and qualitative interviews to recognize signs and symptoms of psychological discomfort and in the use of strategies to minimize such discomfort. This will include reminders that an individual may always choose to not answer any specific question that makes them uncomfortable or to stop surveys and interviews at any time. Referral resources for significant issues will also be made available to study staff should subject referrals to care be desired.

**Ensuring Necessary Medical or Professional Intervention in the Event of Adverse Effects:** Procedures will be put in place for the referral and care, free of charge, for necessary medical or professional intervention in the event of the unlikely occurrence of severe adverse effects. Care will be provided at local Ministry of Health care clinics or nearby referral hospitals as necessary.

## 14.5. Social Impact Events

Individuals enrolled in this study may experience personal problems resulting from the study participation. Such problems are termed *social impact events*. Although study sites will make every effort to protect participant privacy and confidentiality, it is possible that participants' involvement in the study could become known to others, and that participants may experience stigmatization, discrimination or, in rare cases, intimate partner violence as a result of being perceived as being HIV-infected or at risk for HIV infection. For example, participants could be treated unfairly, could have problems being accepted by their families and/or communities, or could experience abuse by their partner. Problems may also occur in circumstances in which study participation is not disclosed, such as impact on employment related to time taken for study visits.

In the event that a participant reports a social impact event, every effort will be made by study staff to provide appropriate assistance, and/or referrals to appropriate resources. Social impact events that are judged by the investigators to be serious, unexpected, or more severe or frequent than anticipated, will be reported to the relevant IRBs.

## 14.6. Benefits

Participants may receive no direct benefit to participation. Participants may benefit from facilitated hypertension treatment, including increased or facilitated access to the clinic or better outcomes for hypertension control related to the service delivery model. From a societal perspective, knowledge gained from this study could improve strategies for improving hypertension treatment and community health.

## 14.7. Compensation

Participants will not receive compensation or payment in this study for taking part in research activities, except those randomized to the travel voucher intervention who link to care within 30 days of screening for the Hypertension Linkage Trial.

## 14.8. Participant Privacy and Confidentiality

Care will be taken to protect the privacy of participants and parents/guardians. Study participants will be identified only by their unique identification number on study documents except those maintained for contact information or consent documents. Participant study documents will be kept in individual files in secure filing cabinets in the study facilities. Testing results and sample and results transport documentation will be maintained in the local labs but will likewise not contain participant identifiers and will be accessed by study personnel only. After the study is over, all files containing personal identifiers such as participants’ names, names of parents, guardians or relatives, phone numbers, and home locations will be destroyed.

In order to ensure data security and integrity, the following measures will be implemented:

- All members of the study team will be educated in the study protocol prior to the onset of the study.
- Detailed Standard Operating Procedures (SOPs) will be written for all project activities and be provided to relevant team members.
- Team members will be thoroughly trained on the SOPs.
- Where applicable, team members will receive additional training on the use of tablet computers.
- All data transcribed from paper will be double data entered or verified.
- All electronic data will be backed up regularly.
- All data will be transferred to the main Data Center in Kampala to the secure server. This sever is backed up on a daily basis and a monthly backup is stored off-site.
- All computers, including the tablets, will be password protected.
- All computers, including tablets, will be locked in a secure room each night.
- Log books and CRFs will be locked in a secure room each night.

## 14.9. Study Discontinuation

The study may be discontinued at any time by the IRB, NIAID, or other government entities as part of their duties to ensure that research participants are protected.

## 14.10. Community Advisory Board and Other Relevant Stakeholders

We will utilize a multilayered set of boards that are part of the SEARCH Sapphire consortium. These include: a) **stakeholder advisory board** consisting of representatives from the Uganda and Kenya Ministries of Health, IDRC, and KEMRI research organizations, in addition to PEPFAR implementing partners at the national and regional level, who will meet after the trial is complete and prior to initiation of additional follow-up studies; b) formal **external scientific advisory board** meeting annually that includes experts in HIV prevention and treatment; experts in PrEP and youth in Uganda and Kenya; and experts in economics/development in East Africa; and c) **in-country advisory board** meeting annually with representation of MoH leads from HIV, non-communicable disease, the World Bank, PEPFAR implementing partners, and persons living with HIV. Finally, **local community advisory boards** will provide invaluable input and communication with biannual meetings. We will have ongoing communication with the Kenya and Uganda National AIDS program, and with the PEPFAR implementing partners.

# 15. STATISTICAL ANALYSIS

## 15.1. Overview

The SEARCH SAPPHIRE Hypertension Linkage Study is an individual randomized controlled trial, designed to test the hypothesis that a transportation voucher and a follow-up phone call will improve linkage to clinic-based hypertension care following community-based screening in three communities in Kenya and Uganda.

Here, we describe the analytic approach for the primary outcomes for the Hypertension Linkage Trial. Analyses of secondary outcomes will be implemented analogously.

##

## 15.2 Individually randomized trial procedures

The community-level inclusion/exclusion criteria, individual-level inclusion/exclusion criteria, recruitment process, study interventions, and study procedures have previously been described. Briefly, consenting participants will be randomized in a 1:1 ratio to the intervention or standard-of-care. Blocked randomization will be stratified on site and further stratified by sex for certain trials enrolling both sexes.

**The primary outcome for the hypertension linkage trial is an indicator of linkage to hypertension care by 30 days.** The primary definition will rely on clinical records; individuals without records will be assumed to never have linked. To assess the impact of the voucher, we will examine, as a secondary outcome, linkage to the scheduled appointment. Other secondary outcomes include blood pressure control (<140/90 mmHg) at 90 days.

We will evaluate the interventions using targeted maximum likelihood estimation (TMLE), which provides precision and power gains over an unadjusted approach (e.g., the Student’s t-test) by adjusting for stratification factors and for chance imbalance between randomized arms on additional baseline predictors of the outcome.^33^ In secondary analyses, we will also implement an unadjusted estimator, the difference of arm-specific average outcomes. In the prevention trials, we will test the null hypothesis of no changes in outcomes due to the intervention with a two-sided test at the 5% significance level. In the treatment and linkage trials, we will test the null hypothesis of no improvements in outcomes due to the intervention with a one-sided test at the 5% significance level. We will also report point estimates and 95% confidence intervals for each effect measure and the arm-specific average outcomes.

###

### 15.2.1 Power calculations

Power and sample size calculations for Phase A individual-level trials were based on standard formulas for a two-sample t-test (dynamic prevention) and two-sample test of proportions (dynamic treatment and linkage), using *power.t.test* and *power.prop.test* in *R*, respectively.^34^ All input parameters were informed by SEARCH data, when possible. We expect these calculations to be conservative, because of the precision gained through stratified randomization and through covariate adjustment during the analysis.^33^

**Figure 1** provides the effect size, on the absolute scale, detected with 80% power varying the number of participants per arm and outcome under the standard-of-care with a two-sided hypothesis test at the 5% significance level.


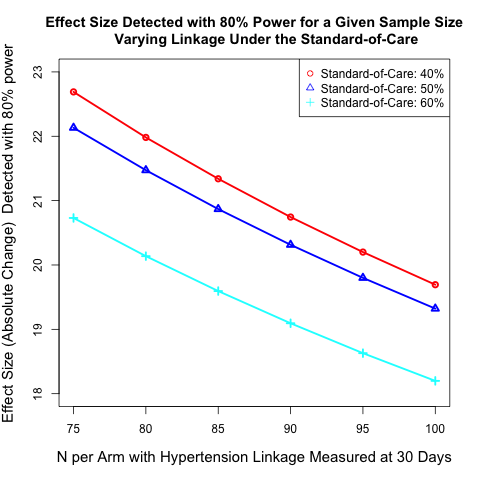


***Figure 1:*** *Effect size (in percent improvement) detected with 80% power for a given sample size and outcome under the standard-of-care for the HTN linkage trial.*

We anticipate having 80% power to detect at least a 19.3% absolute increase in linkage from 50% under the standard-of-care and with 100 participants/arm. Even with 25% fewer participants enrolled (from 100 to 75 participants/arm) and lower or higher linkage under the standard-of-care, these calculations suggest we would be well-powered to detect at least a 22.7% absolute increase in linkage. To ensure sufficient power, we will thus enroll at least 200 participants in the trial.

# 16. PUBLICATION POLICY

The findings from this study may be published in a medical journal. No individual identities will be used in any reports or publications resulting from the study. The researchers will publish results of the study in accordance with NIH, UCSF, UNCST, KEMRI and Makerere University guidelines.

# 17. REFERENCES

1. Gouda HN, Charlson F, Sorsdahl K, et al. Burden of non-communicable diseases in sub-Saharan Africa, 1990-2017: results from the Global Burden of Disease Study 2017. *Lancet Glob Health*. 2019;7(10):e1375-e1387. doi:10.1016/S2214-109X(19)30374-2

2. Geldsetzer P, Manne-Goehler J, Marcus ME, et al. The state of hypertension care in 44 low-income and middle-income countries: a cross-sectional study of nationally representative individual-level data from 1·1 million adults. *Lancet*. 2019;394(10199):652-662. doi:10.1016/S0140-6736(19)30955-9

3. Foreman KJ, Marquez N, Dolgert A, et al. Forecasting life expectancy, years of life lost, and all-cause and cause-specific mortality for 250 causes of death: reference and alternative scenarios for 2016-40 for 195 countries and territories. *Lancet*. 2018;392(10159):2052-2090. doi:10.1016/S0140-6736(18)31694-5

4. Forouzanfar MH, Liu P, Roth GA, et al. Global Burden of Hypertension and Systolic Blood Pressure of at Least 110 to 115 mm Hg, 1990-2015. *JAMA*. 2017;317(2):165-182. doi:10.1001/jama.2016.19043

5. Ataklte F, Erqou S, Kaptoge S, Taye B, Echouffo-Tcheugui JB, Kengne AP. Burden of undiagnosed hypertension in sub-Saharan Africa: a systematic review and meta-analysis. *Hypertension*. 2015;65(2):291-298. doi:10.1161/HYPERTENSIONAHA.114.04394

6. Schutte AE, Srinivasapura Venkateshmurthy N, Mohan S, Prabhakaran D. Hypertension in Low- and Middle-Income Countries. *Circ Res*. 2021;128(7):808-826. doi:10.1161/CIRCRESAHA.120.318729

7. Masenga SK, Hamooya BM, Nzala S, et al. Patho-immune Mechanisms of Hypertension in HIV: a Systematic and Thematic Review. *Curr Hypertens Rep*. 2019;21(7):56. doi:10.1007/s11906-019-0956-5

8. Deeks SG, Phillips AN. HIV infection, antiretroviral treatment, ageing, and non-AIDS related morbidity. *BMJ*. 2009;338:a3172. doi:10.1136/bmj.a3172

9. Nou E, Lo J, Grinspoon SK. Inflammation, immune activation, and cardiovascular disease in HIV. *AIDS*. 2016;30(10):1495-1509. doi:10.1097/QAD.0000000000001109

10. Eyawo O, Brockman G, Goldsmith CH, et al. Risk of myocardial infarction among people living with HIV: an updated systematic review and meta-analysis. *BMJ Open*. 2019;9(9):e025874. doi:10.1136/bmjopen-2018-025874

11. Patel P, Rose CE, Collins PY, et al. Noncommunicable diseases among HIV-infected persons in low-income and middle-income countries: a systematic review and meta-analysis. *AIDS*. 2018;32(Suppl 1):S5-S20. doi:10.1097/QAD.0000000000001888

12. Shah ASV, Stelzle D, Lee KK, et al. Global Burden of Atherosclerotic Cardiovascular Disease in People Living With HIV: Systematic Review and Meta-Analysis. *Circulation*. 2018;138(11):1100-1112. doi:10.1161/CIRCULATIONAHA.117.033369

13. Feinstein MJ, Hsue PY, Benjamin LA, et al. Characteristics, Prevention, and Management of Cardiovascular Disease in People Living With HIV: A Scientific Statement From the American Heart Association. *Circulation*. 2019;140(2):e98-e124. doi:10.1161/CIR.0000000000000695

14. ALLHAT Officers and Coordinators for the ALLHAT Collaborative Research Group. The Antihypertensive and Lipid-Lowering Treatment to Prevent Heart Attack Trial. Major outcomes in high-risk hypertensive patients randomized to angiotensin-converting enzyme inhibitor or calcium channel blocker vs diuretic: The Antihypertensive and Lipid-Lowering Treatment to Prevent Heart Attack Trial (ALLHAT). *JAMA*. 2002;288(23):2981-2997. doi:10.1001/jama.288.23.2981

15. Law MR, Morris JK, Wald NJ. Use of blood pressure lowering drugs in the prevention of cardiovascular disease: meta-analysis of 147 randomised trials in the context of expectations from prospective epidemiological studies. *BMJ*. 2009;338. doi:10.1136/bmj.b1665

16. Bundy JD, Li C, Stuchlik P, et al. Systolic Blood Pressure Reduction and Risk of Cardiovascular Disease and Mortality: A Systematic Review and Network Meta-analysis. *JAMA Cardiol*. 2017;2(7):775-781. doi:10.1001/jamacardio.2017.1421

17. Ojji DB, Mayosi B, Francis V, et al. Comparison of Dual Therapies for Lowering Blood Pressure in Black Africans. *New England Journal of Medicine*. 2019;380(25):2429-2439. doi:10.1056/NEJMoa1901113

18. Chamie G, Hickey MD, Kwarisiima D, Ayieko J, Kamya MR, Havlir DV. Universal HIV Testing and Treatment (UTT) Integrated with Chronic Disease Screening and Treatment: the SEARCH study. *Curr HIV/AIDS Rep*. 2020;17(4):315-323. doi:10.1007/s11904-020-00500-7

19. Kasaie P, Weir B, Schnure M, et al. Integrated screening and treatment services for HIV, hypertension and diabetes in Kenya: assessing the epidemiological impact and cost-effectiveness from a national and regional perspective. *J Int AIDS Soc*. 2020;23 Suppl 1:e25499. doi:10.1002/jia2.25499

20. Kostova D, Spencer G, Moran AE, et al. The cost-effectiveness of hypertension management in low-income and middle-income countries: a review. *BMJ Glob Health*. 2020;5(9). doi:10.1136/bmjgh-2019-002213

21. Shade SB, Osmand T, Kwarisiima D, et al. Costs of integrating hypertension care into HIV care in rural East African clinics. *AIDS*. 2021;35(6):911-919. doi:10.1097/QAD.0000000000002834

22. Brathwaite R, Hutchinson E, McKee M, Palafox B, Balabanova D. The Long and Winding Road: A Systematic Literature Review Conceptualising Pathways for Hypertension Care and Control in Low- and Middle-Income Countries. *Int J Health Policy Manag*. Published online July 18, 2020. doi:10.34172/ijhpm.2020.105

23. Wamai RG, Kengne AP, Levitt N. Non-communicable diseases surveillance: overview of magnitude and determinants in Kenya from STEPwise approach survey of 2015. *BMC Public Health*. 2018;18(3):1224. doi:10.1186/s12889-018-6051-z

24. Kwarisiima D, Balzer L, Heller D, et al. Population-Based Assessment of Hypertension Epidemiology and Risk Factors among. *PLoS One*. 2016;11(5):e0156309. doi:10.1371/journal.pone.0156309

25. Peiris D, Ghosh A, Manne-Goehler J, et al. Cardiovascular disease risk profile and management practices in 45 low-income and middle-income countries: A cross-sectional study of nationally representative individual-level survey data. *PLoS Med*. 2021;18(3):e1003485. doi:10.1371/journal.pmed.1003485

26. Havlir DV, Balzer LB, Charlebois ED, et al. HIV Testing and Treatment with the Use of a Community Health Approach in Rural Africa. *New England Journal of Medicine*. 2019;381(3):219-229. doi:10.1056/NEJMoa1809866

27. Hickey MD, Ayieko J, Owaraganise A, et al. Effect of a patient-centered hypertension delivery strategy on all-cause mortality: Secondary analysis of SEARCH, a community-randomized trial in rural Kenya and Uganda. *PLoS Med*. 2021;18(9):e1003803. doi:10.1371/journal.pmed.1003803

28. Vedanthan R, Kamano JH, DeLong AK, et al. Community Health Workers Improve Linkage to Hypertension Care in Western Kenya. *J Am Coll Cardiol*. 2019;74(15):1897-1906. doi:10.1016/j.jacc.2019.08.003

29. Govindasamy D, Kranzer K, Schaik N van, et al. Linkage to HIV, TB and Non-Communicable Disease Care from a Mobile Testing Unit in Cape Town, South Africa. *PLOS ONE*. 2013;8(11):e80017. doi:10.1371/journal.pone.0080017

30. Kwarisiima D, Atukunda M, Owaraganise A, et al. Hypertension control in integrated HIV and chronic disease clinics in Uganda in the SEARCH study. *BMC Public Health*. 2019;19(1):511. doi:10.1186/s12889-019-6838-6

31. Wierzejska E, Giernaś B, Lipiak A, Karasiewicz M, Cofta M, Staszewski R. A global perspective on the costs of hypertension: a systematic review. *Arch Med Sci*. 2020;16(5):1078-1091. doi:10.5114/aoms.2020.92689

32. Ayieko J, Petersen ML, Charlebois ED, et al. A Patient-Centered Multicomponent Strategy for Accelerated Linkage to Care Following Community-Wide HIV Testing in Rural Uganda and Kenya. *Journal of acquired immune deficiency syndromes (1999)*. 2019;80(4):414-422. doi:10.1097/qai.0000000000001939

33. Van Der Laan M, Rose S. *Targeted Learning: Causal Inference for Observational and Experimental Data*. Springer; 2011.

34. R Core Team. *R: A Language and Environment for Statistical Computing*.; 2019. http://www.R-project.org
